# Comorbidities in primary cicatricial alopecia: a systematic review and meta-analysis

**DOI:** 10.3389/fimmu.2025.1516407

**Published:** 2025-08-29

**Authors:** Tanat Yongpisarn, Kasama Tejapira, Poonkiat Suchonwanit

**Affiliations:** Division of Dermatology, Department of Medicine, Faculty of Medicine Ramathibodi Hospital, Mahidol University, Bangkok, Thailand

**Keywords:** scarring alopecia, lichen planopilaris, frontal fibrosing alopecia, central centrifugal cicatricial alopecia, hair loss, immune-mediated alopecia, associated diseases, autoimmune diseases

## Abstract

**Background:**

Primary cicatricial alopecia (PCA) is known to be associated with various comorbidities; however, findings regarding the likelihood of specific comorbidities in PCA patients have been inconsistent.

**Objective:**

This study aimed to assess the prevalence and odds of specific comorbidities in patients with PCA compared to controls, and to explore the distribution of comorbidities across various types of PCA.

**Methods:**

Electronic searches were conducted using PubMed, Embase, and Scopus from the dates of their inception until July 2024. A total of 116 studies with 33,494 PCA patients that reported data allowing for the calculation of odds ratios (OR) or prevalences of certain comorbidities in PCA patients were included.

**Results:**

Systemic lupus erythematosus is more prevalent among patients with lichen planopilaris (LPP) [OR 3.10 (95% confidence interval: 2.24–4.29), prevalence 2%], frontal fibrosing alopecia (FFA) [OR 6.92 (2.73–17.56), prevalence 5%], and central centrifugal cicatricial alopecia (CCCA) [OR 3.13 (1.03–9.49), prevalence 5%]. Hypothyroidism is more prevalent among patients with LPP [OR of 1.73 (1.24–2.42), prevalence 17%] and FFA [OR 1.86 (1.36–2.55), prevalence 19%]. LPP patients are prone to having dermatological diseases such as atopic dermatitis [OR 3.96 (1.14–13.81), prevalence 9%], lichen planus [OR 19.21 (1.47–251.02), prevalence 8%], psoriasis [OR 4.75 (2.04–11.06), prevalence 3%], and rosacea [OR 4.62 (2.96–7.19), prevalence 5%], while FFA patients are prone to having allergic contact dermatitis [OR 3.19 (1.44–7.08), prevalence 41%] and rosacea [OR 2.37 (1.72–3.29), prevalence 16%]. Coronary artery disease is found to be more common in LPP than controls [OR 1.63 (1.43–1.86), prevalence 8%], while dyslipidemia is more common among FFA [OR 1.41 (1.06–1.88), prevalence 20%] and CCCA [OR 4.46 (1.01–19.75), prevalence 54%] than controls, and diabetes mellitus is more prevalent among CCCA than controls [OR 1.67 (1.03–2.69), prevalence 26%]. While skin cancer [OR 2.22 (1.33–3.70), prevalence 2%] and melanoma [OR 4.46 (1.70–11.76), prevalence 1%] were found to be more common in LPP than controls, rheumatoid arthritis [OR 1.65 (1.09–2.51), prevalence 4%] was found to be more common in FFA than controls, and allergic rhinitis [OR 11.77 (1.55–89.24), prevalence 24%] and anxiety [OR 4.69 (1.29–16.98), prevalence 17%] were found to be more common in CCCA than controls.

**Conclusions:**

Patients with PCA are at higher risk of developing a wide range of comorbidities. Physicians should remain vigilant and conduct thorough investigations when clinical clues are present.

**Systematic Review Registration:**

https://www.crd.york.ac.uk/prospero/display_record.php?RecordID=564852, identifier CRD42024564852.

## 1 Introduction

Cicatricial alopecia, also known as scarring alopecia, results from inflammatory damage to the epithelial stem cells in the follicular bulge region, leading to fibrosis and irreversible hair loss (1). Primary cicatricial alopecia (PCA) occurs when the hair follicle is the primary target of inflammation process, while in secondary cicatricial alopecia, the hair follicle is merely a bystander to the disease process (1). The North American Hair Research Society has proposed classifying PCA into lymphocytic, neutrophilic, mixed, and nonspecific types (2). Lymphocytic PCA encompasses chronic cutaneous lupus erythematosus, classic lichen planopilaris (LPP), frontal fibrosing alopecia (FFA), Graham-Little syndrome, pseudopelade of Brocq (PPB), central centrifugal cicatricial alopecia (CCCA), alopecia mucinosa, and keratosis follicularis spinulosa decalvans (2). Neutrophilic PCA includes folliculitis decalvans (FD) and dissecting cellulitis (DC), while mixed PCA consists of acne keloidalis nuchae (AKN), acne necrotica, and erosive pustular dermatosis (EPD) (2).

Understanding the comorbidities associated with PCA is essential, as they can provide insight into disease etiology and are vital for successful interdisciplinary management. Previous studies on PCA have produced inconsistent findings regarding the prevalence of certain comorbidities and odds of PCA patients having them, likely due to the rarity of PCA and small sample sizes. We aimed to systematically investigate the prevalence of comorbidities and their association with each specific type of PCA, helping guide clinical practice and improve patient care.

## 2 Materials and methods

### 2.1 Study design

The protocol for this analysis was registered in PROSPERO (International Prospective Register of Systematic Reviews; CRD42024564852, https://www.crd.york.ac.uk/prospero/display_record.php?RecordID=564852). The systematic review followed the Preferred Reporting Items for Systematic Reviews and Meta-analyses guidelines (**Supplementary Document**) (3). Electronic searches were conducted from the database’s inception to July 2024 using the PubMed, Embase, and Scopus databases. The search strategy was designed to retrieve all studies on PCA using keywords and a controlled vocabulary. There were no restrictions on the language or publication period in the searches. Only full-text articles were considered, and conference abstracts were excluded due to limited detail and lack of peer review. The search included a combination of terms: ‘primary scarring alopecia’, ‘primary cicatricial alopecia’, ‘lichen planopilaris’, ‘frontal fibrosing alopecia’, ‘Graham Little syndrome’, ‘fibrosing alopecia in a pattern distribution’, ‘pseudopelade of Brocq’, ‘central centrifugal cicatricial alopecia’, ‘discoid lupus erythematosus’, ‘alopecia mucinosa’, ‘keratosis follicularis spinulosa decalvans’, ‘folliculitis decalvans’, ‘dissecting cellulitis’, ‘acne keloidalis’, and ‘erosive pustular dermatosis.’ **Supplementary Table 1** provides details about the search strategy. Grey literature and unpublished data were not considered. If multiple studies utilized patient data from the same database with an overlapping inclusion period, data from the more recent publication or from a case-control study is selected. The comorbid data were not quantitatively utilized if they were reported in a composite manner, such as LPP/FFA or PCA.

### 2.2 Study selection

Two reviewers (TY and KT) independently evaluated each article at both the full-text and title/abstract levels. Full texts of potentially eligible studies were assessed for inclusion. Disagreements regarding the studies’ eligibility were resolved via discussion with a third reviewer (PS). Inclusion criteria were: (1) randomized controlled trials, cohort studies, cross-sectional studies, and case-control studies; (2) studies involving patients with confirmed diagnosis of PCA based on clinical and/or histopathological criteria; (3) studies reporting data allowing calculation of odds ratios (OR) or prevalences of specific comorbidities; (4) full-text articles in any language. Exclusion criteria were: (1) conference abstracts; (2) case reports or case series; (3) studies without clear diagnostic criteria for PCA; (4) studies reporting only composite PCA data without subtype specification. Comorbidities were defined based on clinical diagnosis using ICD codes, physician diagnosis, or validated diagnostic criteria as reported in each study.

### 2.3 Data extraction

Data were extracted from the included studies using a standardized form. The following data were collected: bibliographic data (authors, year of publication), study characteristics (type of study, single or multicenter, study duration, country), alopecia group characteristics (number, age, gender, ethnicity, body mass index (BMI), comorbidity), and treatment information), control group characteristics (number, age, gender, ethnicity, BMI, comorbidity, whether controls were matched for any relevant factors), and comorbidity data.

Corresponding investigators were contacted via email if there was missing data. Two independent reviewers (TY and KT) extracted data, and any discrepancies were discussed and resolved with input from a third reviewer (PS).

### 2.4 Quality assessment

TY and KT independently assessed the quality of descriptive and case-control studies using the Newcastle-Ottawa Scale (NOS) (4). The NOS is a scoring tool comprised of seven items with nine scores that assess how well the investigators selected their participants (score ranges from 0 to 4), the comparability of their results (score ranges from 0 to 2), and the applicability of the outcomes (score ranges from 0 to 3). The higher the score, the higher the study’s quality and the lower the likelihood of bias. Therefore, we classified studies as having high quality if they received a total score of 7 or more, fair quality if they received a score of 4-6, and low quality if they received a score of less than 4. Sensitivity analyses were performed excluding fair-quality studies to assess the robustness of our findings. Meta-analyses were weighted by study quality, with higher-quality studies given greater influence in pooled estimates. Any discrepancies between reviewers regarding the risk of bias in specific studies were resolved through discussion with a third reviewer (PS).

### 2.5 Statistical analysis

A meta-analysis was performed, using an inverse variance method, to pool the ORs of a specific PCA and a specific comorbidity, as well as the prevalences of various comorbidities associated with a specific PCA. Each PCA disorder was analyzed separately. Quantitative analyses were conducted on comorbidity, either OR or prevalence, that were reported in at least two studies; otherwise, they were only analyzed qualitatively.

Heterogeneity was assessed and considered present if a Cochrane Q test p-value was < 0.1 or Higgins *I^2^* ≥ 25% (5). The sources of heterogeneity were explored by fitting each covariate (e.g., age, female gender, and BMI) at a time in a meta-regression model. If the τ^2^ was decreased by ≥ 50% or statistically significant β was revealed, a subgroup analysis was performed based on that covariate if possible (6).

To evaluate publication bias, Deeks funnel plots of the primary outcomes were generated. The Egger linear regression test was applied when a funnel plot suggested possible asymmetry (7). If Egger’s test for a regression intercept gave a p-value < 0.05, a trim and fill method was used to adjust the OR (7). Comprehensive Meta-Analysis software (version 3.3.070, Biostat, Englewood, NJ) was used for all statistical analysis.

## 3 Results

### 3.1 Study characteristics

**Figure 1:**
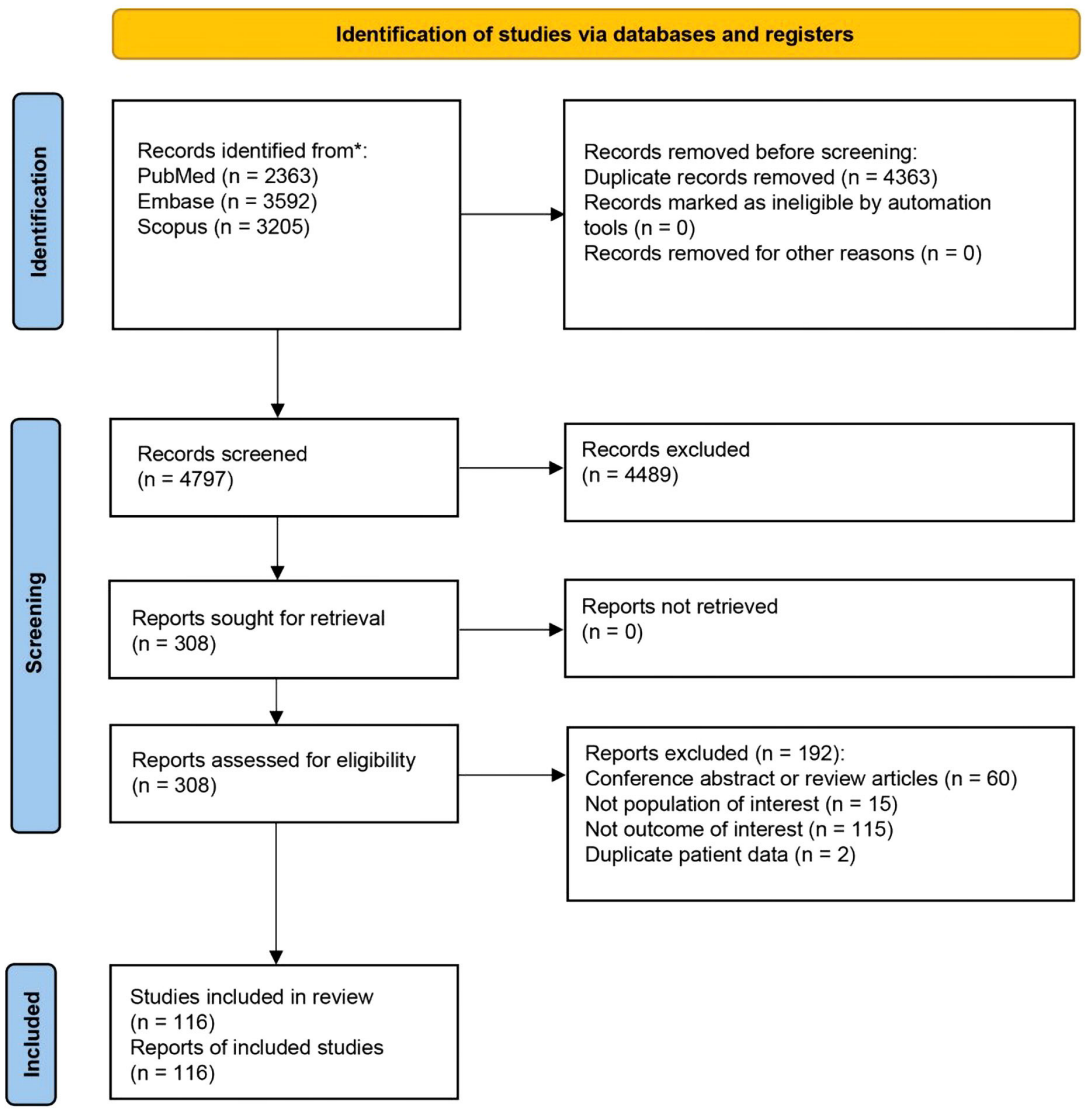
Preferred reporting items for systematic reviews and meta-analyses (PRISMA) flow diagram of search strategy and included studies.

After removing duplicates, 4,797 references were screened by title and abstract. At the full-text stage, 308 full articles met our predefined selection criteria and were sought. We further excluded 192 references for the following reasons: conference abstract or review article (n = 60), not population of interest (i.e., non-alopecia or non-scarring alopecia diagnosis, n = 15), not outcome of interest (i.e., no documented comorbidity prevalence of the patients, n = 115), and duplicate patient data (n = 2) (**Figure 1**). The review included 116 studies, enrolling a total of 33,494 patients with PCA [8,871 LPP patients (8–31), 6,595 FFA patients (12, 16, 19, 21, 23, 25, 27, 30, 32–83), 3,539 CCCA patients (24, 84–99), 157 PPB patients (20), 24 patients with fibrosing alopecia in a pattern distribution (FAPD) (55), 6,158 FD patients (20, 100–104), 4,752 DC patients (20, 105–108), 3,218 AKN patients (109–120), and 180 EPD patients (121–124)] between 2009 and 2024, were included in the review. Characteristic features of the included studies are provided in **Supplementary Tables 2**-**6**.

### 3.2 Lichen planopilaris

#### 3.2.1 Autoimmune diseases

LPP was found to be significantly associated with systemic lupus erythematosus (SLE) [OR 3.10 (95% confidence interval: 2.24–4.29), prevalence 2%]. Inflammatory bowel diseases [OR 2.49 (0.91–6.77), prevalence 5%], including Crohn’s disease [OR 0.75 (0.37–1.49), prevalence 1%] and ulcerative colitis [OR 1.23 (0.32–4.74), prevalence 2%], were not statistically associated with LPP, as were other autoimmune diseases, such as ankylosing spondylitis [OR 1.07 (0.83–1.39), prevalence 1%], celiac disease [OR 1.06 (0.34–3.26), prevalence 2%], multiple sclerosis [OR 1.76 (0.65–4.74), prevalence <1%], rheumatoid arthritis [OR 1.59 (0.90–2.78), prevalence 3%], Sjogren’s syndrome [OR 1.24 (0.81–1.89), prevalence 1%], and type 1 diabetes mellitus [OR 0.77 (0.26–2.27), prevalence 1%].

#### 3.2.2 Cardiovascular and metabolic diseases

Coronary artery disease was found to be more common in LPP than controls [OR 1.63 (1.43–1.86), prevalence 8%]. However, we did not find a statistically significant association between LPP and cardiovascular diseases in general [OR 0.74 (0.48–1.16), prevalence 10%] or other cardiovascular comorbidities like congestive heart failure [OR 0.61 (0.15–2.44), prevalence 4%], myocardial infarction [OR 1.27 (0.87–1.86), prevalence 2%], peripheral artery disease [OR 1.03 (0.68–1.58), prevalence 5%], and stroke [0.57 (0.11–2.84), prevalence 3%]. Interestingly, Kim et al. have reported an adjusted hazard ratio (aHR) of 1.93 (1.07–3.49) for coronary heart disease in their cohort of LPP/FFA patients, while aHR of 1.18 (0.69–2.04) and 0.60 (0.23–1.60) were found for cardiovascular disease and stroke, respectively (125).

None of the metabolic diseases, such as diabetes mellitus [OR 0.87 (0.52–1.47), prevalence 10%], dyslipidemia [OR 1.36 (0.64–2.92), prevalence 27%], hypertension [OR 1.21 (0.63–2.33), prevalence 23%], and obesity [OR 1.82 (0.51–6.45), prevalence 20%], were found to be significantly associated with LPP.

#### 3.2.3 Dermatologic diseases

Lichen planus was found to be strongly associated with LPP [OR 19.21 (1.47–251.02), prevalence 8%]. Other dermatological diseases such as atopic dermatitis [OR 3.96 (1.14–13.81), prevalence 9%], psoriasis [OR 4.75 (2.04–11.06), prevalence 3%], and rosacea [OR 4.62 (2.96–7.19), prevalence 5%] were also found to be significantly associated with LPP. Skin cancer [OR 2.22 (1.33–3.70), prevalence 2%] and melanoma [OR 4.46 (1.70–11.76), prevalence 1%] were also found to be more prevalent in LPP than controls. For alopecia areata (AA), however, the association was not statistically significant [OR 2.56 (0.26–25.76), prevalence 3%].

#### 3.2.4 Thyroid disorders

Thyroid diseases, in general, were found to be associated with LPP [OR 2.64 (1.13–6.21), prevalence 24%]. A statistically significant association was found between LPP and hypothyroidism [OR 1.73 (1.24–2.42), prevalence 17%], hyperthyroidism [OR 1.17 (1.00–1.36), prevalence 1%], and thyroiditis [OR 1.21 (1.00–1.46), prevalence 6%]. Although a statistically significant association was found between Hashimoto thyroiditis [OR 4.24 (1.04–17.24), prevalence 7%] and LPP, a publication bias was identified (p=0.014, Egger’s test). After to a trim and fill analysis to account for the publication bias, the association was no longer statistically significant [adjusted OR 1.72 (0.54–5.41)].

#### 3.2.5 Other diseases

Allergic rhinitis [OR 1.05 (0.50–2.19), prevalence 12%], anxiety [OR 1.19 (0.94–1.51), prevalence 22%], asthma [OR 0.91 (0.53–1.56), prevalence 8%], depression [OR 0.85 (0.45–1.61), prevalence 15%], and vitamin D deficiency [OR 0.91 (0.33–2.54), prevalence 19%] were not statistically associated with LPP.

**Figure 2:**
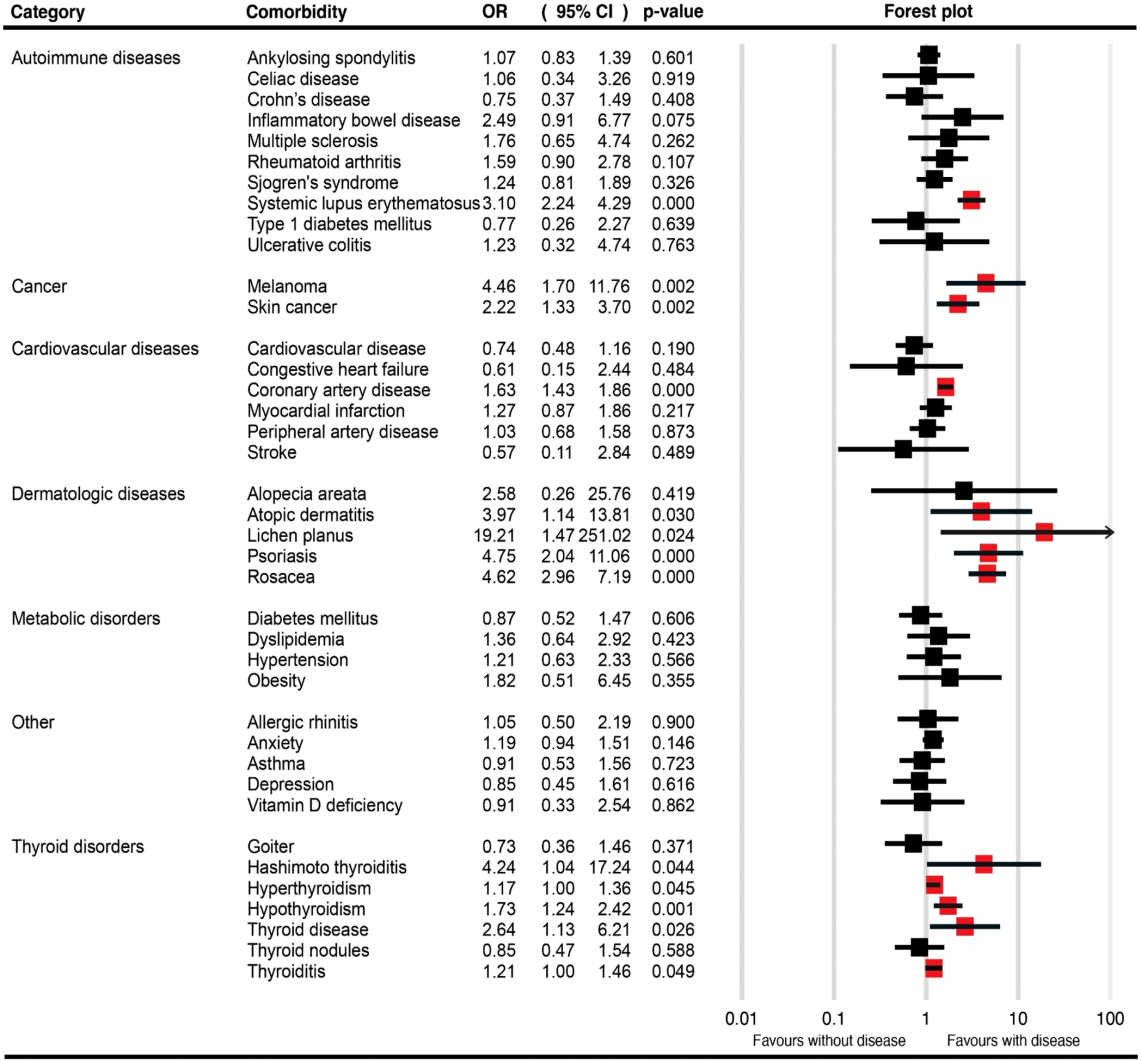
Forest plots for the pooled odds ratio of various comorbidities in patients with lichen planopilaris.

**Table 1** provides a summary of the pooled OR for the described comorbidities of LPP patients, along with the associated pooled prevalences. **Figure 2**, **Supplementary Figure 1** depict the pooled OR and prevalence of the comorbidities in patients with LPP, respectively.

**Table 1:** Summary of the pooled odds ratio and pooled prevalences of the comorbidities of lichen planopilaris, frontal fibrosing alopecia, and central centrifugal cicatricial alopecia patients.

PCA subtype	Comorbidity	Odds ratio (95% CI)	p-value	No. of studies	PCA	Control	*I^2^*	Egger’s test	Prevalence (95% CI)	No. of studies
LPP	Allergic rhinitis	1.05 (0.50–2.19)	0.900	3 (9, 12, 17)	2447	40921	78.31	0.648	0.12 (0.01–0.59)	5 (9, 12, 17, 23, 29)
	Alopecia areata	2.58 (0.26–25.76)	0.419	2 (12, 14)	319	517	60.29	–	0.03 (0.02–0.06)	4 (12, 14, 23, 30)
	Ankylosing spondylitis	1.07 (0.83–1.39)	0.601	2 (14, 17)	2026	40520	0.00	–	0.01 (0–0.13)	2 (14, 17)
	Anxiety	1.19 (0.94–1.51)	0.146	4 (9, 13, 14, 17)	2734	41360	48.69	0.763	0.22 (0.14–0.33)	8 (9, 13, 14, 17, 22, 23, 28, 29)
	Asthma	0.91 (0.53–1.56)	0.723	2 (9, 17)	2360	40598	62.33	–	0.08 (0.02–0.28)	4 (9, 17, 28, 29)
	Atopic dermatitis	3.97 (1.14–13.81)	0.030	3 (9, 13, 17)	2502	41166	90.81	0.976	0.09 (0.03–0.23)	5 (9, 13, 17, 28, 29)
	Cardiovascular disease	0.74 (0.48–1.16)	0.190	3 (12, 14, 15)	527	725	21.51	0.497	0.1 (0.01–0.51)	3 (12, 14, 15)
	Celiac disease	1.06 (0.34–3.26)	0.919	3 (9, 14, 19)	770	1189779	0.00	0.21	0.02 (0.01–0.03)	5 (9, 14, 19, 28, 29)
	Congestive heart failure	0.61 (0.15–2.44)	0.484	2 (14, 17)	2258	40714	87.74	–	0.04 (0.03–0.05)	2 (14, 17)
	Coronary heart disease	1.63 (1.43–1.86)	<0.001	2 (10, 14)	3402	63442194	0.00	–	0.08 (0.07–0.09)	5 (10, 14, 22, 27, 28)
	Crohn’s disease	0.75 (0.37–1.49)	0.408	2 (12, 17)	2113	40843	0.00	–	0.01 (0–0.02)	3 (12, 17, 28)
	Depression	0.85 (0.45–1.61)	0.616	4 (9, 12–14)	795	1163	77.81	0.413	0.15 (0.08–0.25)	9 (9, 12–14, 22, 23, 28–30)
	Diabetes mellitus	0.87 (0.52–1.47)	0.606	7 (8–10, 12, 13, 17, 19)	6129	64673077	96.38	0.232	0.1 (0.07–0.13)	13 (8–10, 12, 13, 17, 19, 22, 23, 26, 27, 29, 30)
	Dyslipidemia	1.36 (0.64–2.92)	0.423	9 (8–15, 17)	6552	63484028	99.19	0.271	0.27 (0.21–0.33)	15 (8–15, 17, 22, 23, 27–30)
	Goiter	0.73 (0.36–1.46)	0.371	3 (8, 9, 14)	732	353	0.00	0.121	0.03 (0.02–0.05)	3 (8, 9, 14)
	Hashimoto thyroiditis	4.24 (1.04–17.24)	0.044	3 (8, 9, 19)	704	1189666	0.00	0.014	0.07 (0.04–0.11)	7 (8, 9, 19, 23, 28, 29, 31)
	Hypertension	1.21 (0.63–2.33)	0.566	7 (10–15, 17)	6052	63483869	98.74	0.302	0.23 (0.18–0.3)	14 (10–15, 17, 22, 23, 26–30)
	Hyperthyroidism	1.17 (1.00–1.36)	0.045	7 (8, 9, 12–14, 17, 19)	3191	1231271	0.00	0.875	0.01 (0–0.05)	7 (8, 9, 12–14, 17, 19)
	Hypothyroidism	1.73 (1.24–2.42)	0.001	9 (8, 9, 12–15, 17–19)	3425	1231509	67.03	0.205	0.17 (0.12–0.22)	13 (8, 9, 12–15, 17–19, 21, 26, 28, 29)
	Inflammatory bowel disease	2.49 (0.91–6.77)	0.075	2 (13, 19)	346	1190075	66.36	–	0.05 (0.01–0.19)	2 (13, 19)
	Lichen planus	19.21 (1.47–251.02)	0.024	2 (17, 19)	2230	1230027	96.09	–	0.08 (0.04–0.16)	5 (17, 19, 21, 29, 30)
	Melanoma	4.46 (1.70–11.76)	0.002	2 (13, 17)	2168	41088	0.00	–	0.01 (0–0.05)	4 (13, 17, 28, 29)
	Multiple sclerosis	1.76 (0.65–4.74)	0.262	4 (12, 14, 17, 19)	2549	1230544	13.51	0.043	0 (0–0.01)	4 (12, 14, 17, 19)
	Myocardial infarction	1.27 (0.87–1.86)	0.217	2 (10, 17)	5196	63482520	75.88	–	0.02 (0.02–0.04)	3 (10, 17, 23)
	Obesity	1.82 (0.51–6.45)	0.355	3 (9, 10, 13)	3646	63442646	97.70	0.034	0.2 (0.14–0.27)	7 (9, 10, 13, 22, 26, 28, 29)
	Peripheral artery disease	1.03 (0.68–1.58)	0.873	2 (14, 17)	2258	40714	14.50	–	0.05 (0.01–0.32)	3 (14, 17, 22)
	Psoriasis	4.75 (2.04–11.06)	<0.001	5 (9, 12, 14, 17, 19)	2793	1230996	70.53	0.864	0.03 (0.01–0.08)	8 (9, 12, 14, 17, 19, 23, 28, 29)
	Rheumatoid arthritis	1.59 (0.90–2.78)	0.107	5 (9, 12, 13, 17, 19)	2938	1230867	45.78	0.91	0.03 (0.01–0.08)	7 (9, 12–14, 17, 19, 28)
	Rosacea	4.62 (2.96–7.19)	<0.001	2 (13, 14)	374	762	0.00	–	0.05 (0.02–0.15)	5 (13, 14, 23, 28, 29)
	Sjogren’s syndrome	1.24 (0.81–1.89)	0.326	4 (9, 14, 17, 19)	2796	1230299	0.00	0.741	0.01 (0.01–0.03)	6 (9, 14, 17, 19, 28, 29)
	Skin cancer	2.22 (1.33–3.70)	0.002	2 (14, 17)	2258	40714	5.38	–	0.02 (0–0.77)	2 (14, 17)
	Stroke	0.57 (0.11–2.84)	0.489	2 (14, 17)	2258	40714	89.84	–	0.03 (0.01–0.09)	3 (14, 17, 23)
	SLE	3.10 (2.24–4.29)	<0.001	5 (9, 12, 13, 17, 19)	2793	1228996	0.00	0.977	0.02 (0.01–0.04)	7 (9, 12, 13, 17, 19, 23, 28)
	Thyroid disease	2.64 (1.13–6.21)	0.026	4 (8, 15, 16, 18)	428	350	69.24	0.414	0.24 (0.17–0.31)	8 (8, 15, 16, 18, 22, 27, 28, 30)
	Thyroid nodules	0.85 (0.47–1.54)	0.588	2 (9, 14)	566	272	0.00	–	0.02 (0.01–0.07)	5 (8, 9, 14, 28, 29)
	Thyroiditis	1.21 (1.00–1.46)	0.049	2 (14, 17)	2258	40714	0.00	–	0.06 (0.05–0.07)	2 (14, 17)
	Type 1 diabetes mellitus	0.77 (0.26–2.27)	0.639	3 (13, 14, 19)	578	1190269	0.00	0.462	0.01 (0.01–0.03)	5 (13, 14, 19, 28, 31)
	Ulcerative colitis	1.23 (0.32–4.74)	0.763	2 (9, 17)	2360	40598	62.77	–	0.02 (0.01–0.05)	3 (9, 17, 28)
	Vitamin D deficiency	0.91 (0.33–2.54)	0.862	2 (9, 17)	2360	40598	91.84	–	0.19 (0.04–0.55)	5 (9, 17, 24, 26, 28)
	Vitiligo	2.41 (0.94–6.20)	0.069	4 (9, 12, 13, 19)	767	1190476	0.00	0.578	0.01 (0.01–0.03)	9 (9, 12, 13, 19, 23, 26, 28–30)
FFA	Allergic contact dermatitis	3.19 (1.44–7.08)	0.004	2 (36, 42)	44	492	0.00	–	0.41 (0.09–0.83)	2 (36, 42)
	Allergic rhinitis	0.86 (0.65–1.13)	0.278	2 (12, 41)	570	774	0.00	–	0.04 (0–0.41)	3 (12, 23, 41)
	Breast cancer	1.61 (0.42–6.13)	0.483	2 (35, 38)	412	555	71.99	–	0.05 (0.03–0.1)	7 (30, 35, 38, 51, 53, 71, 83)
	Depression	1.22 (0.88–1.69)	0.228	2 (12, 41)	570	774	0.00	–	0.13 (0.06–0.24)	6 (12, 23, 30, 41, 54, 58)
	Dyslipidemia	1.41 (1.05–1.88)	0.020	4 (12, 37, 39, 41)	719	914	11.77	0.375	0.2 (0.15–0.27)	20 (12, 23, 27, 30, 37, 39, 41, 43, 44, 50, 52, 54, 57, 58, 64, 65, 69, 70, 77, 80)
	Diabetes mellitus	1.03 (0.72–1.47)	0.862	5 (12, 19, 37, 39, 41)	893	1190421	0.00	0.253	0.07 (0.05–0.11)	17 (12, 19, 23, 27, 30, 37, 39, 41, 50, 53, 54, 57, 58, 65, 69, 74, 77)
	Hypertension	1.79 (0.85–3.79)	0.128	4 (12, 37, 39, 41)	719	914	83.15	0.233	0.27 (0.21–0.34)	24 (12, 23, 27, 30, 37, 39, 41, 43, 44, 48, 50, 52–54, 57, 58, 64, 65, 69, 70, 74, 77, 79, 80)
	Hyperthyroidism	1.11 (0.36–3.41)	0.855	3 (12, 19, 38)	601	1190177	0.00	0.479	0.02 (0.01–0.04)	7 (12, 19, 38, 57, 70, 77, 79)
	Hypothyroidism	1.86 (1.36–2.55)	<0.001	4 (12, 19, 34, 38)	631	1190207	0.00	0.897	0.19 (0.15–0.24)	24 (12, 19, 21, 34, 38, 44–46, 48, 50, 52–54, 57–60, 68–70, 74, 76, 80, 83)
	Lupus	2.42 (0.86–6.87)	0.096	3 (36, 38, 41)	783	1266	0.00	0.229	0.01 (0–0.04)	3 (36, 38, 41)
	Multiple sclerosis	0.84 (0.19–3.62)	0.811	2 (12, 19)	293	1189830	0.00	–	0.01 (0–0.03)	2 (12, 19)
	Psoriasis	2.11 (0.32–13.73)	0.436	3 (12, 19, 41)	744	1190281	93.01	0.791	0.04 (0.03–0.05)	18 (12, 19, 23, 41, 45, 46, 51, 53, 54, 57, 58, 65, 70, 74, 76, 79, 80, 83)
	Rheumatoid arthritis	1.65 (1.08–2.51)	0.019	4 (12, 19, 38, 41)	1052	1190628	0.00	0.136	0.04 (0.02–0.07)	9 (12, 19, 38, 41, 44, 45, 54, 57, 80)
	Rosacea	2.37 (1.72–3.28)	<0.001	3 (38, 39, 41)	858	838	0.00	0.132	0.16 (0.1–0.23)	20 (23, 38, 39, 41, 48, 49, 54, 57, 59, 60, 64–68, 71–73, 76, 80)
	SLE	6.92 (2.73–17.56)	<0.001	3 (12, 19, 34)	323	1187860	0.00	0.888	0.05 (0.03–0.08)	10 (12, 19, 23, 34, 43, 45, 54, 58, 74, 83)
	Type 1 diabetes mellitus	0.39 (0.07–2.16)	0.279	2 (19, 38)	482	1189854	0.00	–	0.01 (0–0.01)	4 (19, 38, 54, 57)
	Thyroid disease	1.66 (1.21–2.28)	0.002	3 (16, 37, 41)	536	582	0.00	0.474	0.26 (0.2–0.33)	15 (16, 27, 30, 37, 41, 49, 54, 57, 58, 65, 74, 78, 82, 83)
	Vitiligo	4.01 (1.95–8.26)	<0.001	5 (12, 19, 37, 38, 41)	1102	1190728	13.93	0.47	0.03 (0.02–0.04)	17 (12, 19, 23, 30, 37, 38, 41, 45, 46, 49, 54, 57, 58, 65, 74, 80, 83)
CCCA	Acne	2.21 (0.31–15.52)	0.427	2 (87, 88)	105	436	46.28	–	0.4 (0.11–0.78)	3 (87, 88, 98)
	Allergic rhinitis	11.77 (1.55–89.24)	0.017	2 (86, 88)	254	413	56.14	–	0.24 (0.06–0.63)	2 (86, 88)
	Anxiety	4.69 (1.29–16.98)	0.019	3 (85, 86, 88)	407	566	73.46	0.932	0.17 (0.03–0.62)	3 (85, 86, 88)
	Asthma	7.43 (0.85–64.69)	0.069	2 (86, 88)	254	413	59.31	–	0.17 (0.04–0.51)	2 (86, 88)
	Atopic dermatitis	1.06 (0.05–22.69)	0.972	2 (86, 87)	253	425	90.11	–	0.06 (0.04–0.1)	2 (86, 87)
	Depression	1.93 (0.65–5.70)	0.236	2 (85, 86)	354	354	85.55	–	0.26 (0.05–0.71)	2 (85, 86)
	Diabetic mellitus	1.67 (1.03–2.69)	0.036	7 (85–88, 90–92)	1355	41447	87.97	0.586	0.26 (0.19–0.34)	13 (85–88, 90–98)
	Dyslipidemia	4.46 (1.01–19.75)	0.049	3 (85, 86, 88)	407	566	96.01	0.686	0.54 (0.35–0.71)	5 (85, 86, 88, 94, 97)
	Hirsutism	2.83 (0.35–22.70)	0.328	3 (85, 87, 88)	258	589	73.09	0.022	0.09 (0.01–0.51)	3 (85, 87, 88)
	Hypertension	1.62 (0.78–3.34)	0.195	5 (85, 86, 88, 90, 92)	908	1943	92.04	0.438	0.64 (0.55–0.73)	6 (85, 86, 88, 90, 92, 94)
	Leiomyoma	2.23 (0.90–5.52)	0.084	4 (84, 85, 90, 92)	1101	488187	94.36	0.978	0.15 (0.08–0.27)	4 (84, 85, 90, 92)
	Obesity	0.80 (0.28–2.30)	0.676	4 (85, 88, 91, 92)	1028	40926	96.08	0.707	0.72 (0.37–0.92)	4 (85, 88, 91, 92)
	Seborrheic dermatitis	1.16 (0.74–1.83)	0.521	3 (85, 87, 88)	258	589	4.83	0.061	0.28 (0.15–0.45)	5 (85, 87, 88, 96, 98)
	SLE	3.13 (1.03–9.49)	0.044	2 (85, 88)	206	365	0.00	–	0.05 (0.02–0.13)	2 (85, 88)
	Vitiligo	12.14 (0.49–302.35)	0.128	2 (85, 88)	53	212	0.00	–	0.01 (0–0.04)	2 (85, 88)

Summary of the pooled odds ratio and pooled prevalences of the comorbidities of lichen planopilaris, frontal fibrosing alopecia, and central centrifugal cicatricial alopecia patients.

CCCA, central centrifugal cicatricial alopecia; FFA, frontal fibrosing alopecia; LPP, lichen planopilaris; PCA, primary cicatricial alopecia; SLE, systemic lupus erythematosus.

### 3.3 Frontal fibrosing alopecia

#### 3.3.1 Autoimmune diseases

The results showed a strong association between SLE [OR 6.92 (2.73–17.56), prevalence 5%] and FFA. We also discovered a statistical association between FFA and rheumatoid arthritis [OR 1.65 (1.08–2.51), prevalence 4%], but not with multiple sclerosis [OR 0.84 (0.19–3.62), prevalence 4%] or type 1 diabetes mellitus [OR 0.39 (0.07–2.16), prevalence 1%].

#### 3.3.2 Dermatologic diseases

Allergic contact dermatitis [OR 3.19 (1.44–7.08), prevalence 41%] and rosacea [OR 2.37 (1.72–3.28), prevalence 16%] were found to be associated with FFA; however, for psoriasis [OR 2.11 (0.32–13.73), prevalence 4%], the association was not statistically significant.

#### 3.3.3 Metabolic disorders

Dyslipidemia [OR 1.41 (1.05–1.88), prevalence 20%] was found to be associated with FFA, while diabetes mellitus [OR 1.03 (0.72–1.47), prevalence 7%] and hypertension [OR 1.79 (0.85–3.79), prevalence 27%] were not statistically associated with FFA.

#### 3.3.4 Thyroid disorders

Thyroid disease in general [OR 1.66 (1.21–2.28), prevalence 26%] was found to have statistical association with FFA; however, only hypothyroidism [OR 1.86 (1.36–2.55), prevalence 19%] was statistically associated with FFA, not hyperthyroidism [OR 1.11 (0.36–3.41), prevalence 2%].

#### 3.3.5 Other diseases

Breast cancer [OR 1.61 (0.42–6.13), prevalence 5%], allergic rhinitis [OR 0.86 (0.65–1.13), prevalence 4%], and depression [OR 1.22 (0.88–1.69), prevalence 13%] were not statistically associated with FFA.

**Figure 3:**
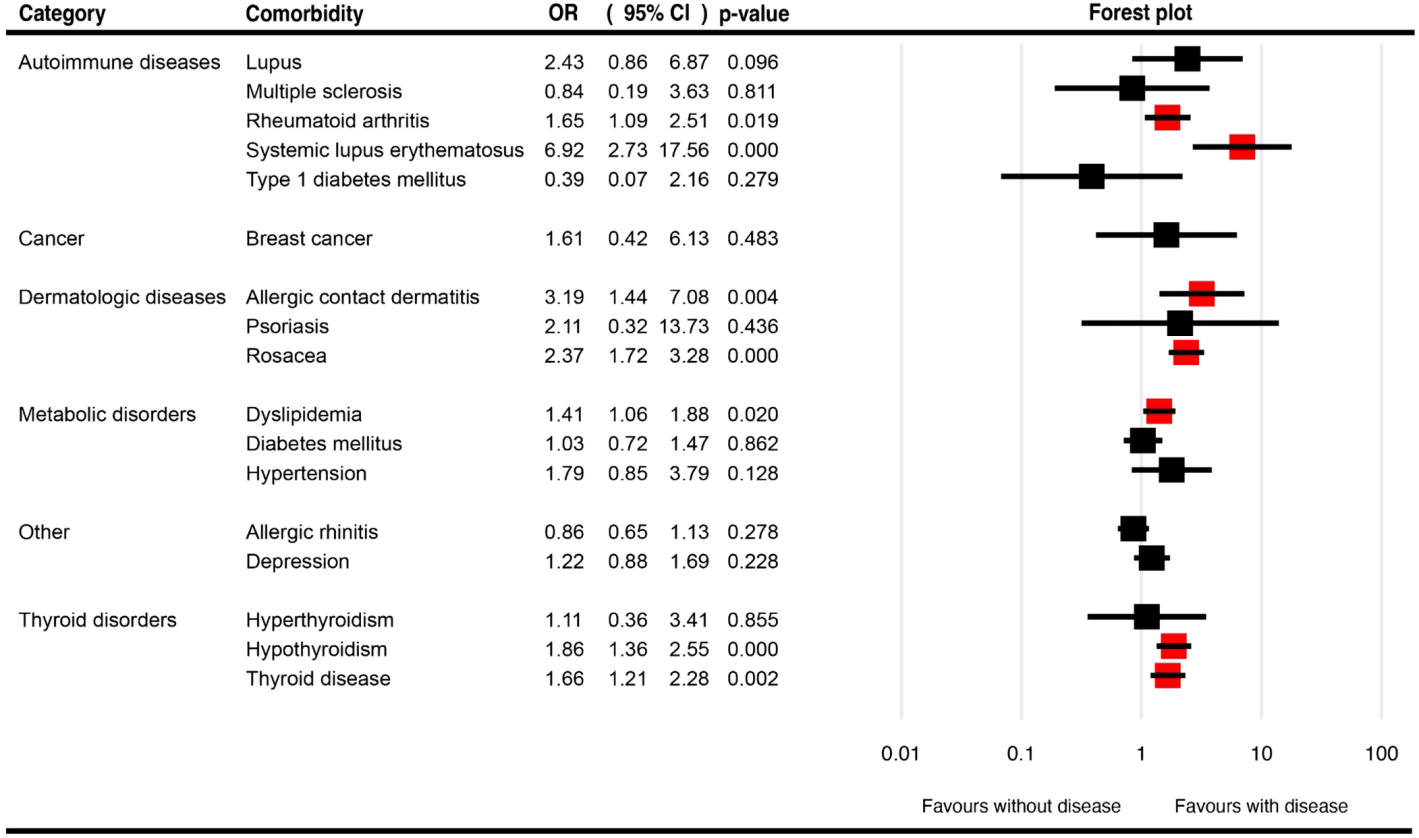
Forest plots for the pooled odds ratio of various comorbidities in patients with frontal fibrosing alopecia.

**Table 1** summarizes the pooled OR for the described comorbidities of FFA patients and the associated pooled prevalences. **Figure 3**, **Supplementary Figure 2** present the pooled OR and prevalence of the comorbidities in patients with FFA, respectively.

### 3.4 Central centrifugal cicatricial alopecia

#### 3.4.1 Autoimmune diseases

SLE [OR 3.13 (1.03–9.49), prevalence 5%] was found to have statistically significant association with CCCA.

#### 3.4.2 Dermatologic diseases

Acne [OR 2.21 (0.31–15.52), prevalence 40%], atopic dermatitis [OR 1.06 (0.05–22.69), prevalence 6%], and seborrheic dermatitis [OR 1.16 (0.74–1.83), prevalence 28%] were not found to be statistically associated with CCCA.

#### 3.4.3 Metabolic disorders

Dyslipidemia [OR 4.46 (1.01–19.75), prevalence 54%] was found to have a strong statistical association with CCCA, while diabetes mellitus [OR 1.67 (1.03–2.69), prevalence 26%] was also statistically associated with CCCA; however, other metabolic diseases such as hypertension [OR 1.62 (0.78–3.34), prevalence 64%], obesity [OR 0.80 (0.28–2.30), prevalence 72%], and hirsutism [OR 2.83 (0.35–22.70), prevalence 9%] were not statistically associated with CCCA.

#### 3.4.4 Other diseases

Allergic rhinitis [OR 11.77 (1.55–89.24), prevalence 24%] and anxiety [OR 4.69 (1.29–16.98), prevalence 17%] were strongly associated with CCCA. Asthma [OR 7.43 (0.85–64.69), prevalence 17%], depression [OR 1.93 (0.65–5.70), prevalence 26%], and leiomyoma [OR 2.23 (0.90–5.52), prevalence 15%] were not associated with CCCA with statistical significance.

**Figure 4:**
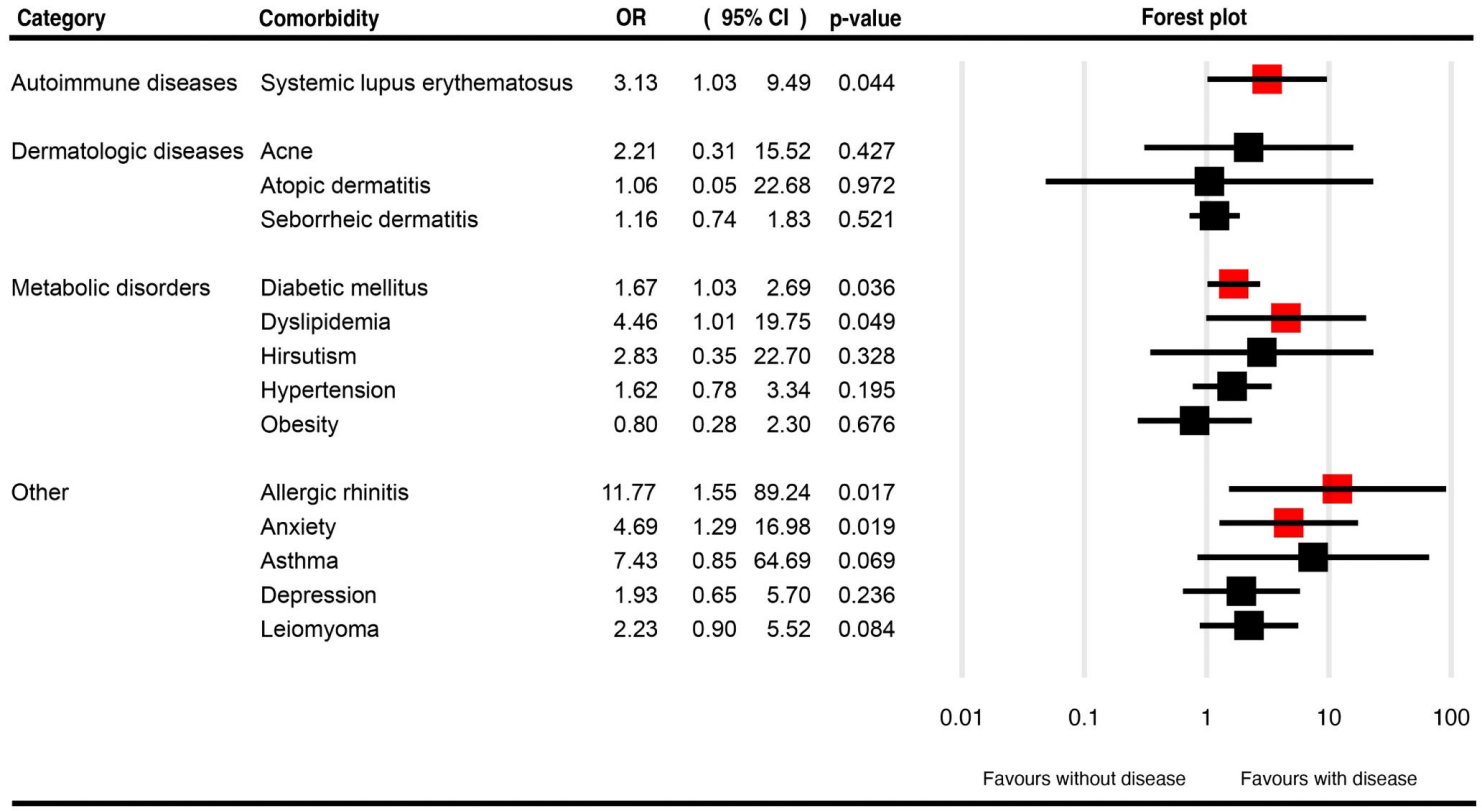
Forest plots for the pooled odds ratio of various comorbidities in patients with central centrifugal cicatricial alopecia.

**Table 1** presents a summary of the pooled OR for the described comorbidities of CCCA patients, as well as the associated pooled prevalences. **Figure 4**, **Supplementary Figure 3** illustrate the pooled OR and prevalence of the comorbidities in patients with CCCA, respectively.

### 3.5 Other primary cicatricial alopecia disorders

Among 24 FAPD patients, we identified AA [prevalence 12.5%], celiac disease [prevalence 8.3%], rheumatoid arthritis [prevalence 8.3%], frontal fibrosing alopecia [prevalence 8.3%], pernicious anemia [prevalence 4.2%], scalp psoriasis [prevalence 4.2%], and traction alopecia [prevalence 4.2%]. For AKN, common comorbidities include acne [prevalence 29%], acne conglobata [prevalence 14%], FD [prevalence 21%], and pseudofolliculitis barbae [prevalence 26%]. Acne conglobata [prevalence 24%] is also prevalent in DC, and a higher prevalence of hidradenitis suppurativa was found for DC [prevalence 26%], compared to FD [prevalence 4%] and AKN [prevalence 3%]. And for EPD, patients often report actinic keratosis [prevalence 21%], basal cell carcinoma [prevalence 7%], and squamous cell carcinoma [prevalence 13%] as their comorbidities. **Supplementary Figures 4**-**7** show forest plots for the pooled prevalence of the comorbidities in patients with FD, DC, AKN, and EPD, respectively.

### 3.6 Quality assessment

**Supplementary Table 7** provides a summary of the quality assessment scores for comparative and descriptive studies included in the review. The average quality assessment score was 7.78 (range: 4–9), with 101 high-quality and 15 fair-quality studies. No studies were classified as low-quality. The most common quality concerns in fair-quality studies were related to the comparability of cohorts and adequacy of follow-up. Sensitivity analyses excluding the 15 fair-quality studies did not substantially alter our main findings, with all significant associations remaining statistically significant.

### 3.7 Meta-regression and subgroup analysis

Meta-regression was performed for all analyses that were found to have significant heterogeneity and did not find any potential source of heterogeneity, except for one analysis in which we found that female proportion seems to play a role in the odds of depression in the LPP cohorts (**Supplementary Figure 8**). However, we chose not to perform a subgroup analysis due to the limited number of studies that included a narrow range of female proportions, specifically from 72.4 to 91.5%.

## 4 Discussion

This meta-analysis reveals distinct comorbidity patterns across PCA. The high prevalence and diversity of comorbidities suggest these conditions have systemic implications. Specifically, LPP showed strong associations with autoimmune disorders, particularly thyroid disease and lichen planus. FFA demonstrated links to both autoimmune and metabolic comorbidities, whereas CCCA exhibited significant associations with metabolic and hormonal factors. These distinct patterns underscore the complex, multifactorial nature of PCA and suggest they may be manifestations of broader systemic dysregulation rather than isolated scalp disorders. The elevated ORs for specific comorbidities emphasize the importance of a comprehensive approach to managing patients with these conditions, as they are likely at higher risk for multiple systemic conditions.

### 4.1 Autoimmune diseases

Statistically significantly higher odds of SLE were found among those with lymphocytic PCA, particularly LPP, FFA, and CCCA, compared with controls. Peroxisome proliferator-activated receptor-γ (PPAR-γ), a nuclear hormone receptor, is involved in the regulation of inflammation and tissue repair (126). It has been proposed to be the link between LPP and SLE (12), as PPAR-γ has been implicated in the pathogenesis of both PCA and SLE (126, 127). Specifically, PPAR-γ expression is altered in both conditions, potentially contributing to dysregulated immune responses and tissue damages (126, 128–130). PPAR-γ is also involved in the pathogenesis of rheumatoid arthritis (131), which we found to have a significant association with FFA. Therefore, further studies exploring the link between PCA and various autoimmune diseases are needed.

The association between PCA and multiple autoimmune conditions aligns with the concept of Multiple Autoimmune Syndrome (MAS), where at least three autoimmune diseases coexist in the same patient. Tatu and Ionescu described a case of MAS type 3, characterized by the coexistence of autoimmune thyroiditis, vitiligo, and AA (132). Although their report focused on non-scarring alopecia, the principle of shared autoimmune pathophysiology may extend to primary cicatricial alopecias, suggesting that PCA could represent a cutaneous manifestation within a broader autoimmune context.

### 4.2 Thyroid disorders

Similar to a previous meta-analysis, we also found a statistically significant association between LPP and hypothyroidism (133). In addition, we also found FFA to be associated with hypothyroidism, which can be observed in nearly one fifth of patients with either LPP or FFA. The association between thyroid disorders, especially hypothyroidism, and PCA could be explained by the prevalent immune-mediated hypothyroidism, such as Hashimoto thyroiditis. We believe hypothyroidism should be considered when evaluating LPP or FFA patients, given the well-established link between thyroid diseases and autoimmune skin diseases (134). This association underscores the importance of routine thyroid function screening in patients diagnosed with these forms of PCA, especially in cases with clinical suspicion. However, the need for thyroid investigation in asymptomatic patients remains controversial.

### 4.3 Dermatologic diseases

Compared to previous meta-analysis, we found a similar association and prevalence of rosacea in FFA (135). We also find that rosacea is strongly associated with LPP, although the prevalence is lower than in FFA. Apart from rosacea, LPP is also strongly linked to lichen planus and other immune-mediated skin diseases, including atopic dermatitis and psoriasis.

Although the evidence is limited to two studies, allergic contact dermatitis is also significantly associated with FFA. Given that FFA association with skin care products has been shown previously (136), it may be sensible to inform patients of the risk.

In contrast to other autoimmune skin diseases such as vitiligo and AA (137–140), we found a higher risk of skin cancer and melanoma among LPP patients. Actinic keratosis and non-melanocytic skin cancer are also found to be prevalent among EPD. Given the increased risk of skin cancer and dermatologic diseases, we believe a detailed full-body dermatological examination for patients with PCA is necessary.

### 4.4 Metabolic disorders

We have confirmed the findings of a previous meta-analysis that dyslipidemia is not statistically associated with LPP (141). Additionally, we also did not identify any association between LPP and other metabolic diseases, including diabetes mellitus, hypertension, and obesity. However, we did find an association between FFA and dyslipidemia, while CCCA is associated with both dyslipidemia and diabetes mellitus. These associations suggest a potential role of metabolic inflammation in their pathogenesis (126). Further studies exploring the association between PCA and metabolic disorders are needed.

### 4.5 Cardiovascular diseases

Kim et al. have previously found an association between PCA and incident cardiovascular disease, particularly coronary heart disease (125), which is also evident in our LPP findings. Certain interleukins (IL), especially IL-18 (142), are associated with cardiovascular risk, which makes autoimmune patients more prone to coronary diseases. We believe this issue requires further investigation and physicians should be on high alert for coronary events.

### 4.6 Other diseases

Similar to a previous meta-analysis (143), evidence is limited for vitamin D deficiency and PCA, and evidence so far suggests that vitamin D deficiency is not statistically associated with LPP. In addition, the lack of statistical association between uterine leiomyoma and CCCA is noted in our study, while strong associations between allergic rhinitis, anxiety, and CCCA were found. We suspect that the high prevalence of uterine leiomyoma may not be directly related to the disease. Regular psychosocial assessment and support is crucial for patients with CCCA as well as other PCAs. Further studies exploring the relationship between allergic rhinitis and CCCA are needed.

### 4.7 Histopathological integration and mechanistic insights

Recent histopathological studies have identified variations in inflammatory patterns across PCA subtypes that may explain their different comorbidity profiles. The predominantly CD8+ T-cell-mediated inflammation in LPP and FFA contrasts with the CD4+ T-cell-predominant mixed inflammatory infiltrate seen in CCCA, potentially explaining their distinct associations with autoimmune and metabolic conditions, respectively (144–146).

The association between PCA and various systemic conditions reflects complex shared pathogenic mechanisms involving inflammatory and immune-mediated pathways. The histopathological hallmark of lymphocytic PCA, destruction of follicular stem cells in the bulge region with perifollicular fibrosis, mirrors inflammatory patterns seen in associated autoimmune conditions. This parallel extends to the molecular level, where PPAR-γ signaling emerges as a crucial link between PCA and systemic disorders.

In LPP and FFA, decreased PPAR-γ expression in hair follicle stem cells triggers proinflammatory cytokine production and subsequent stem cell loss. This mechanism shows striking similarity to PPAR-γ dysfunction in SLE, where impaired PPAR-γ signaling contributes to immune dysregulation and tissue damage (126). The strong association between these conditions likely reflects this shared pathogenic pathway. For CCCA, the significant association with metabolic disorders may similarly reflect common inflammatory mechanisms. PPAR-γ’s role in regulating both immune responses and metabolic homeostasis provides a potential molecular explanation for these associations (147). The presence of metabolic inflammation in conditions such as diabetes and dyslipidemia may exacerbate the follicular inflammation characteristic of CCCA.

FAPD has been recently recognized as a subtype of LPP that presents with a pattern mimicking androgenetic alopecia (148). Histologically, FAPD exhibits a lymphocytic scarring process similar to classic LPP, although detailed characterization of the lymphocyte subtypes in its inflammatory infiltrate remains lacking. While its recognition has expanded our understanding of how scarring alopecia can clinically manifest, data regarding its associated systemic conditions remain limited.

### 4.8 Clinical significance of risk estimates

The magnitude of associations found in this meta-analysis has important clinical implications. A 3-fold increased risk of SLE in LPP patients translates to an absolute risk of approximately 2%, compared to 0.65% in the general population. While this remains a relatively low absolute risk, it warrants clinical vigilance given SLE’s potential severity. More practically significant is the 17-19% prevalence of hypothyroidism in LPP and FFA, a frequency that justifies routine screening.

The 8% prevalence of coronary artery disease in LPP patients (OR 1.63) represents a clinically meaningful increase that should prompt cardiovascular risk assessment and aggressive management of modifiable risk factors. Similarly, the high prevalence of dyslipidemia in CCCA (54%) and diabetes mellitus (26%) necessitates proactive metabolic screening and intervention in this population.

### 4.9 Clinical implications and screening recommendations

Based on our findings, we recommend a comprehensive screening approach for PCA patients. Initial assessment should include thyroid function tests and thyroid antibody screening for all PCA patients. Autoimmune screening with antinuclear antibody testing should be considered in patients presenting with systemic symptoms. Metabolic screening including lipid profile and fasting glucose is particularly important in FFA and CCCA patients. Annual full-body skin examinations are warranted in LPP patients due to their increased skin cancer risk.

The complexity of comorbidities in PCA necessitates multidisciplinary care coordination. Establishing collaborative relationships with endocrinology for thyroid management, rheumatology for suspected autoimmune conditions, and maintaining regular dermatologic surveillance are essential components of comprehensive care. Mental health support should be integrated into the management plan, given the significant psychological burden associated with PCA. This coordinated approach ensures that the multiple systemic manifestations of PCA are adequately addressed, ultimately improving patient outcomes and quality of life.

### 4.10 Limitations

This study has some limitations. Firstly, some comorbidities were infrequently reported and thus were not able to be quantitatively analyzed. Additionally, there is an inadequate number of cohort studies that document incident cases of various comorbidities for quantitative analysis. Furthermore, significant statistical heterogeneity was found in some of our analyses, and despite meta-regression analysis, we were unable to identify the source of heterogeneity, except for one analysis. This is partly due to the limited number of available studies. Potential sources of heterogeneity include variations in diagnostic criteria for PCA subtypes, differences in study populations (such as ethnicity, age distribution, geographic location, disease severity, and comorbidity burden), methodological differences across studies, differences in healthcare systems and access to specialist care, and temporal changes in awareness and screening practices for both PCA and associated conditions. These factors highlight the need for cautious interpretation of pooled estimates for these specific associations. While we employed appropriate statistical methods, the adjusted results may still be influenced by unreported negative findings.

## 5 Conclusions

This meta-analysis significantly contributes to our understanding of PCA by providing a comprehensive overview of associated comorbidities. PCA patients are at an increased risk of developing a variety of comorbidities, such as SLE, hypothyroidism, metabolic diseases, and various dermatologic conditions. The findings emphasize the need for a multidisciplinary approach to patient care and highlight the importance of considering PCA as potentially systemic disorders with localized scalp manifestations. Therefore, clinicians should maintain a high level of vigilance for comorbid conditions in PCA patients and conduct investigations when clinical signs are observed. Future research should focus on elucidating the mechanisms behind these associations through prospective cohort studies, which would help establish causality and determine whether interventions targeting comorbidities improve outcomes in PCA. While limitations such as study heterogeneity and the challenge of establishing causality exist, this analysis provides valuable insights that can guide clinical practice and future investigations in PCA.

## Data Availability

The original contributions presented in this study are included in the article/**Supplementary Material**, further inquiries can be directed to the corresponding author.

## Supplementary material

Supplementary Figure 1 Forest plots for the pooled prevalence of the comorbidities in patients with lichen planopilaris.

Supplementary Figure 2 Forest plots for the pooled prevalence of the comorbidities in patients with frontal fibrosing alopecia.

Supplementary Figure 3 Forest plots for the pooled prevalence of the comorbidities in patients with central centrifugal cicatricial alopecia.

Supplementary Figure 4 Forest plots for the pooled prevalence of the comorbidities in patients with folliculitis decalvans.

Supplementary Figure 5 Forest plots for the pooled prevalence of the comorbidities in patients with dissecting cellulitis.

Supplementary Figure 6 Forest plots for the pooled prevalence of the comorbidities in patients with acne keloidalis nuchae

Supplementary Figure 7 Forest plots for the pooled prevalence of the comorbidities in patients with erosive pustular dermatosis of the scalp

Supplementary Figure 8 Bubble plot for the meta-regression analysis of the pooled odds ratio of depression in patients with lichen planopilaris, using the female proportion as a covariate.
