# Supplementary figures and images for: Comorbidities in primary cicatricial alopecia: a systematic review and meta-analysis
Source: Front Immunol. 2025 Aug 29;16:1516407. doi: 10.3389/fimmu.2025.1516407 (PMC12426186; doi:10.3389/fimmu.2025.1516407)

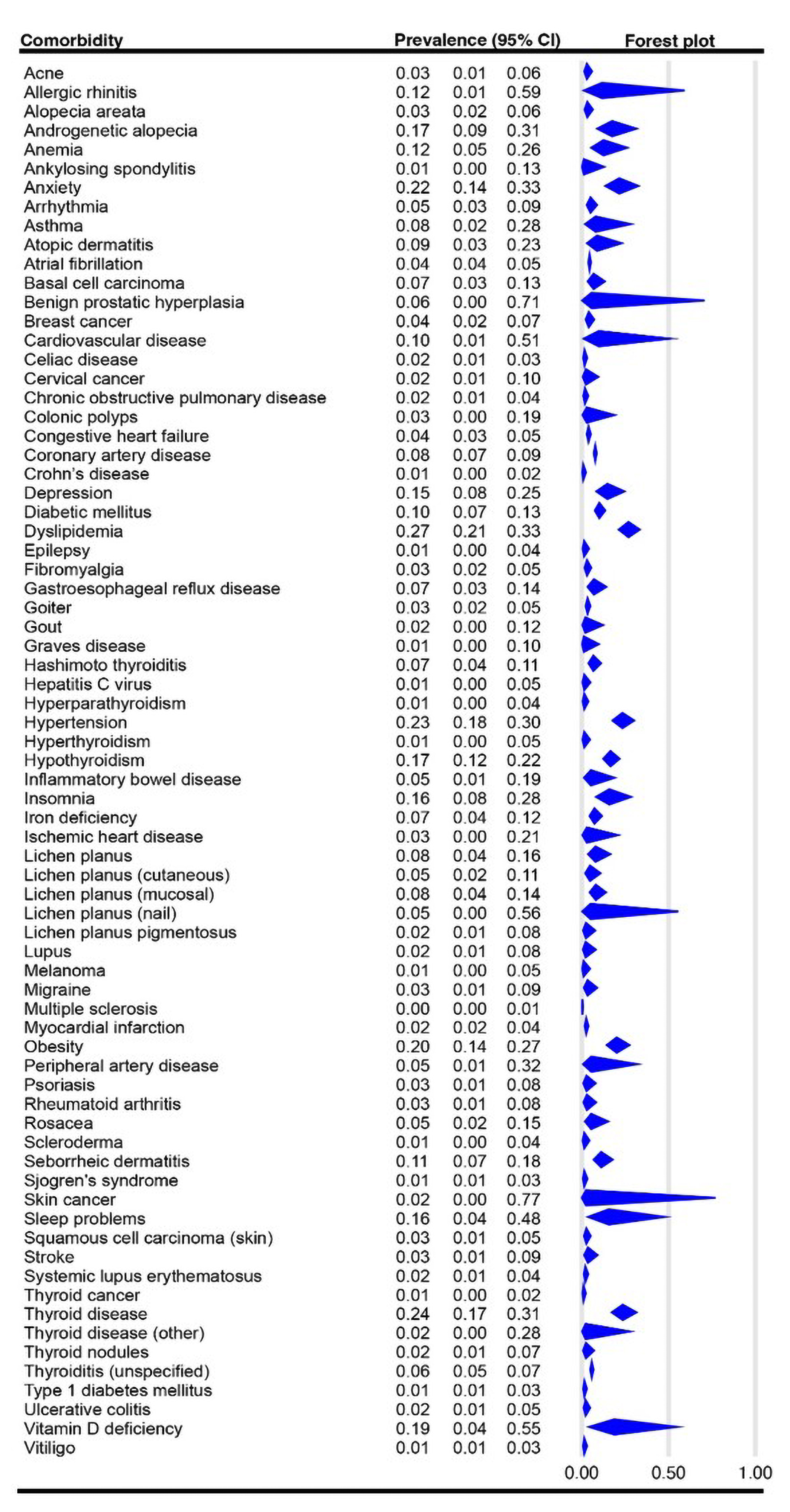

Supplement: Supplementary Figure 1 — Forest plots for the pooled prevalence of the comorbidities in patients with lichen planopilaris. [file Image1.jpeg]

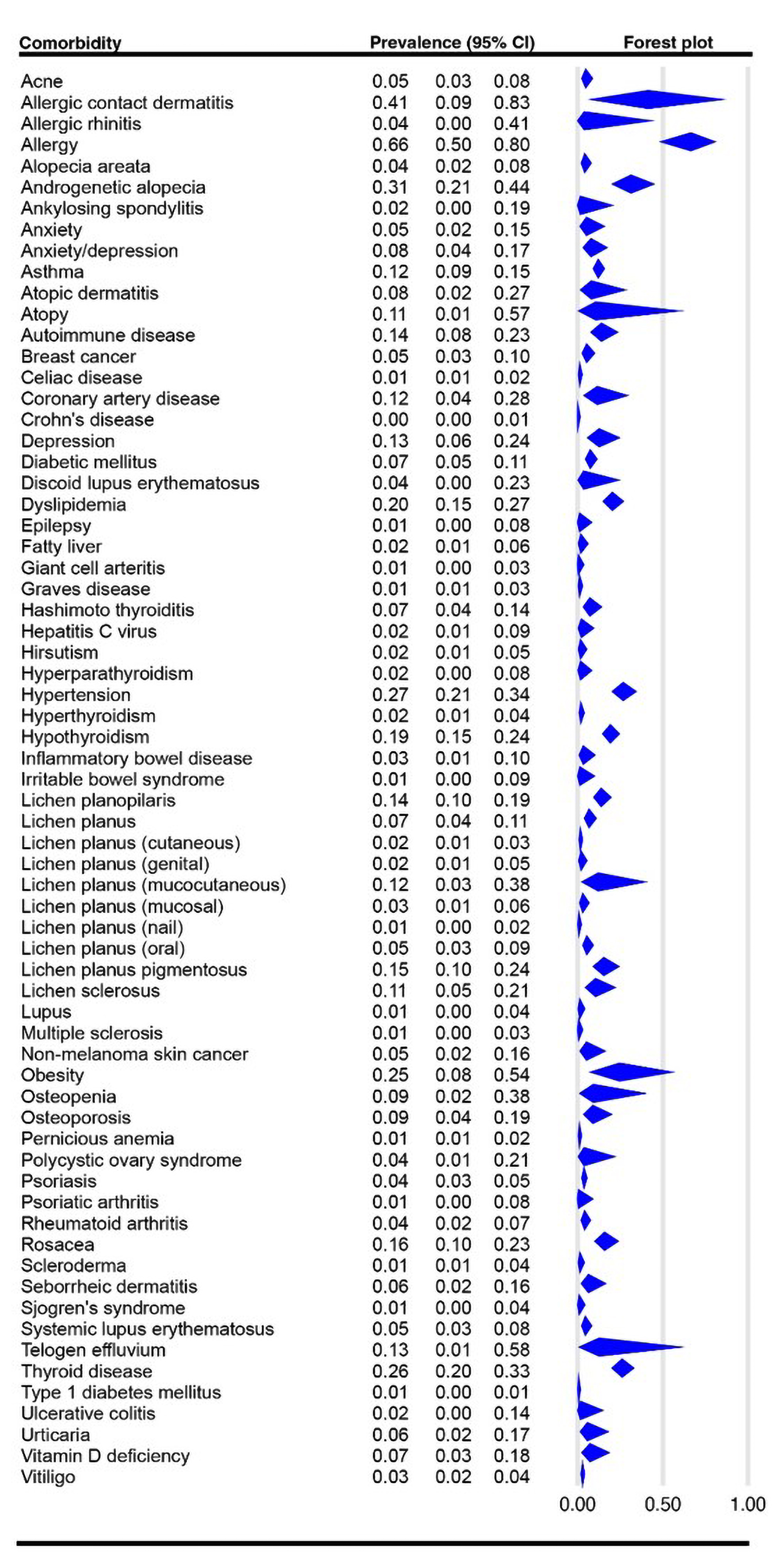

Supplement: Supplementary Figure 2 — Forest plots for the pooled prevalence of the comorbidities in patients with frontal fibrosing alopecia. [file Image2.jpeg]

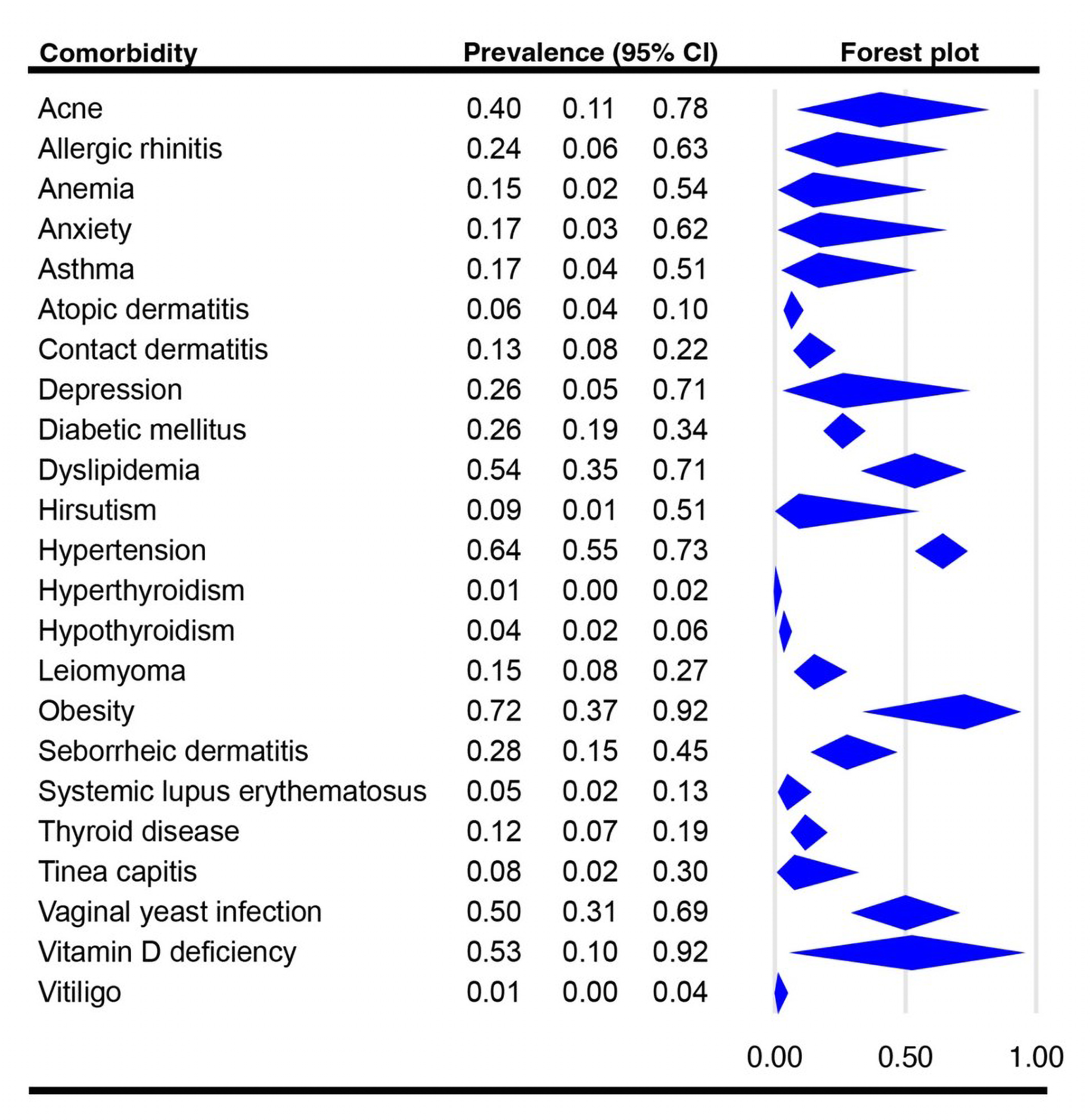

Supplement: Supplementary Figure 3 — Forest plots for the pooled prevalence of the comorbidities in patients with central centrifugal cicatricial alopecia. [file Image3.jpeg]

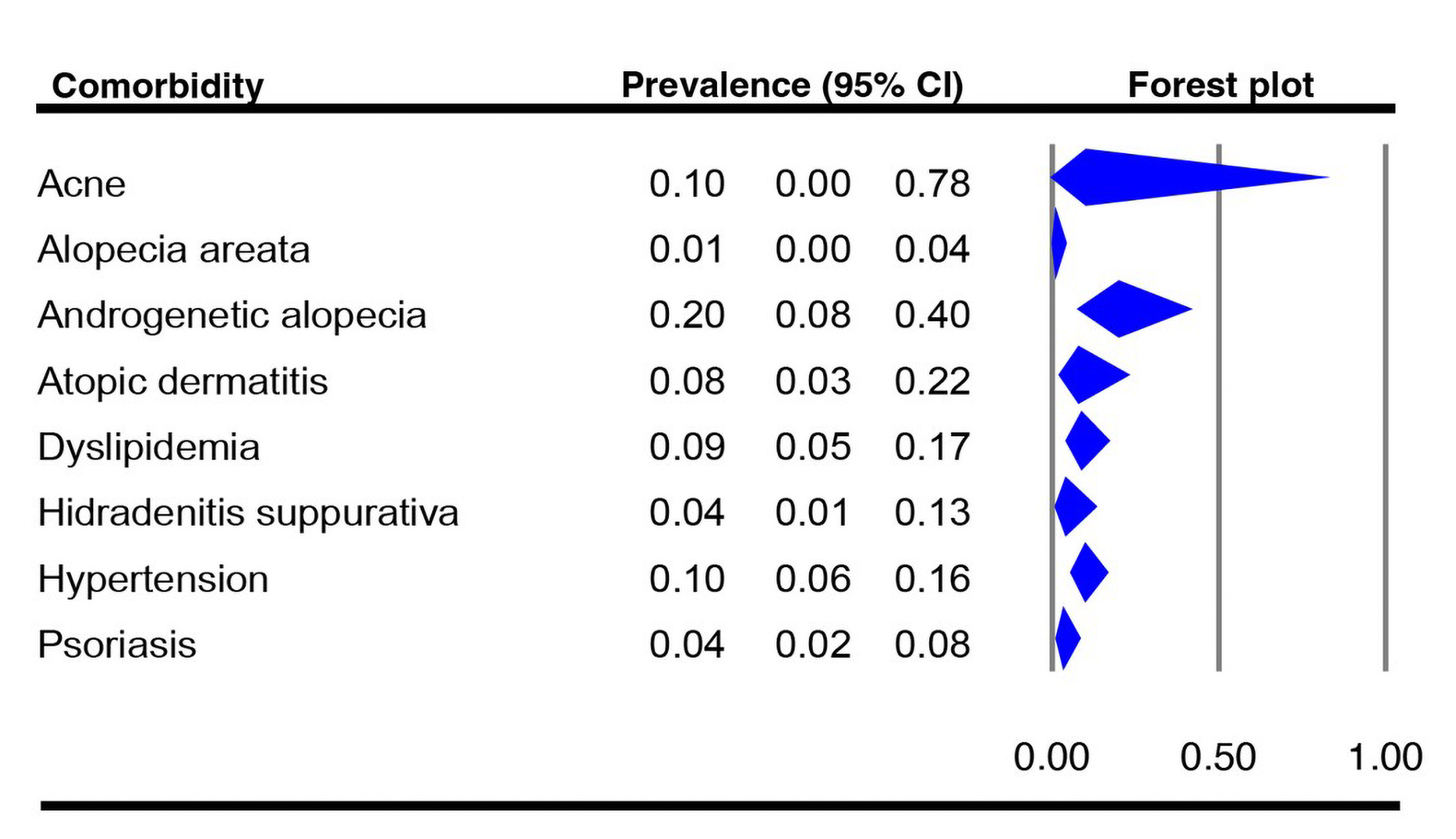

Supplement: Supplementary Figure 4 — Forest plots for the pooled prevalence of the comorbidities in patients with folliculitis decalvans. [file Image4.jpeg]

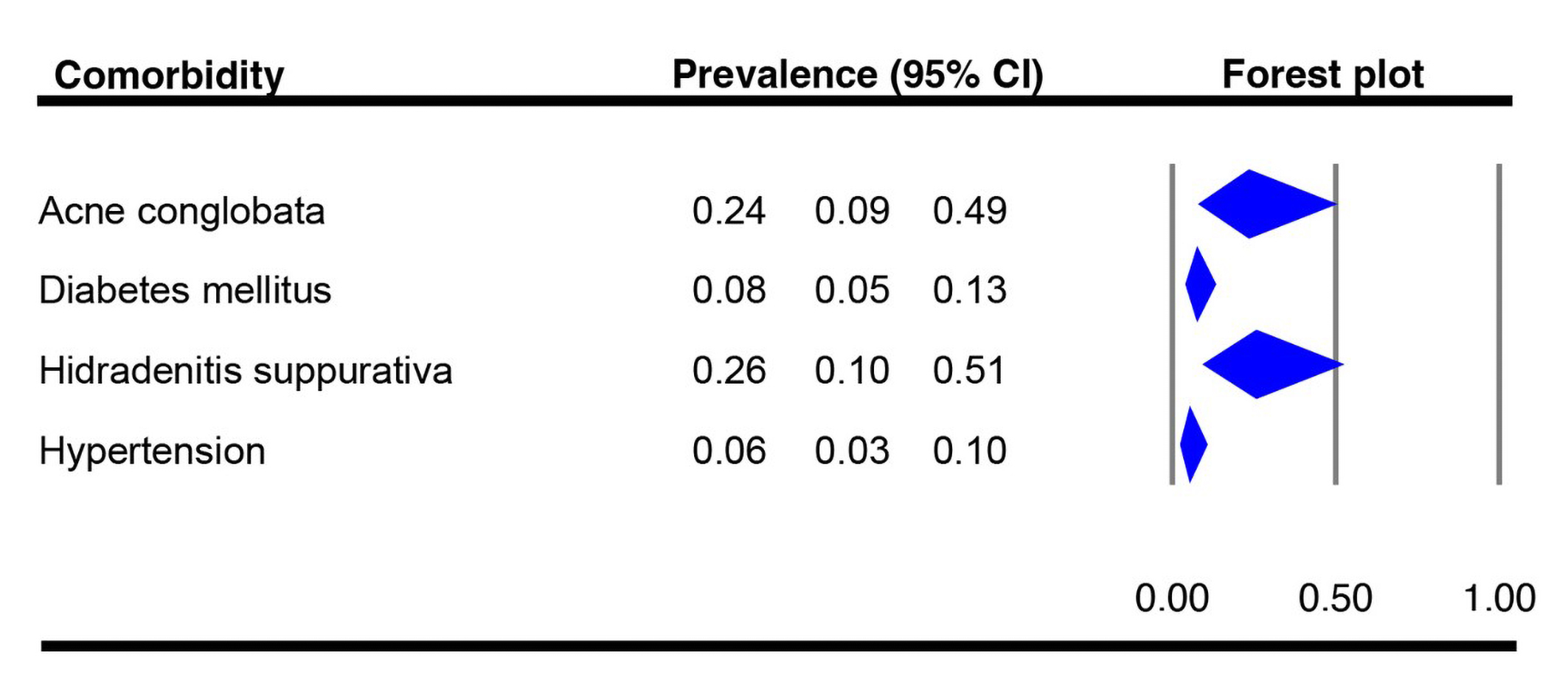

Supplement: Supplementary Figure 5 — Forest plots for the pooled prevalence of the comorbidities in patients with dissecting cellulitis. [file Image5.jpeg]

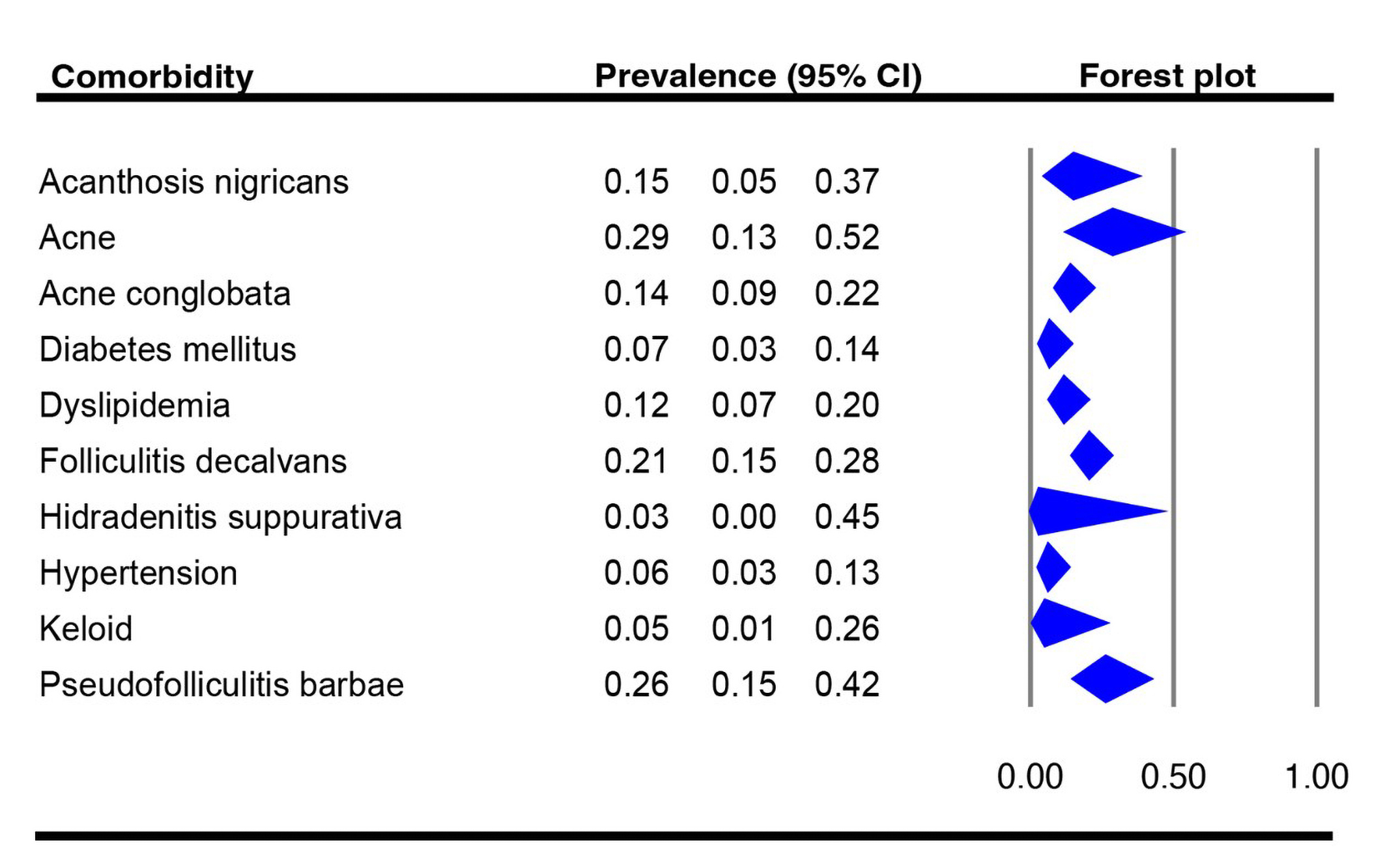

Supplement: Supplementary Figure 6 — Forest plots for the pooled prevalence of the comorbidities in patients with acne keloidalis nuchae [file Image6.jpeg]

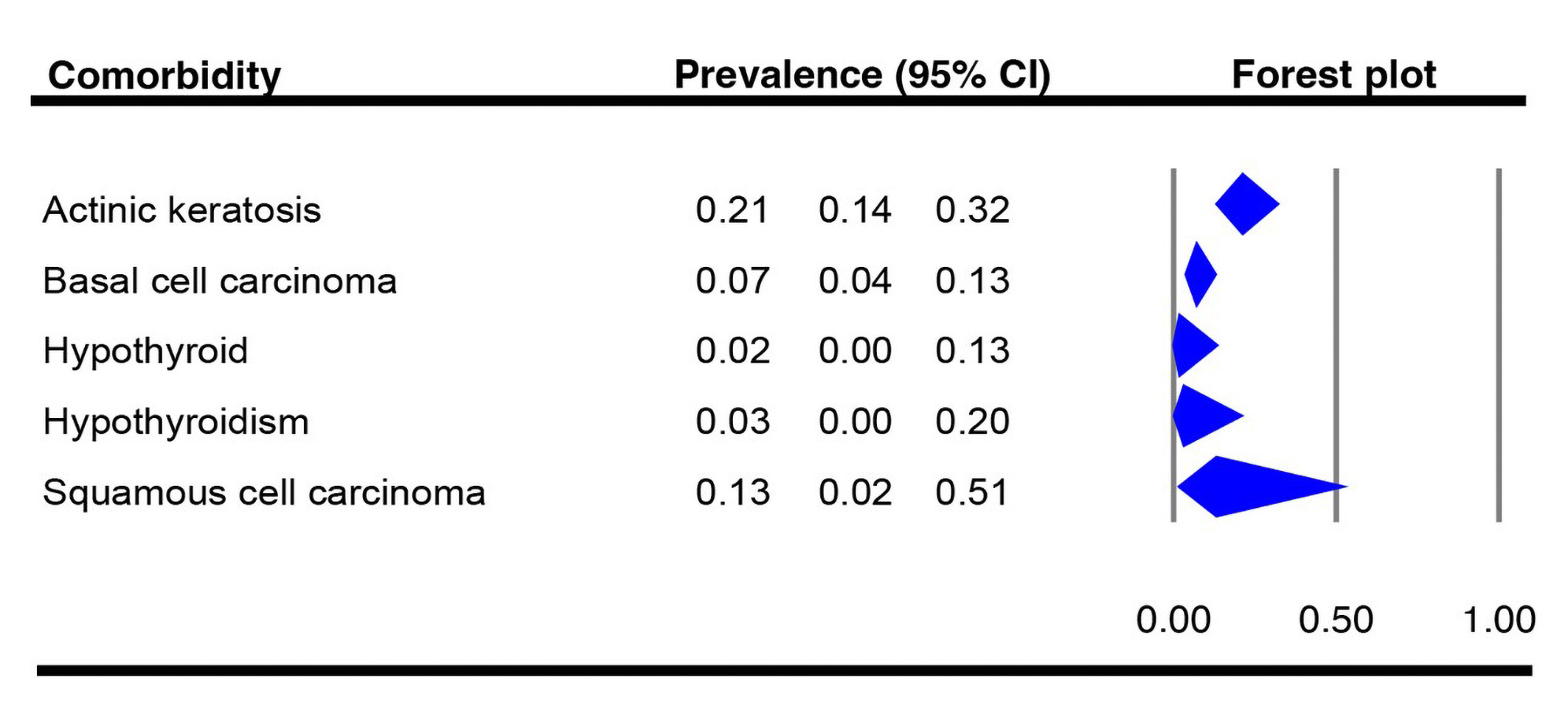

Supplement: Supplementary Figure 7 — Forest plots for the pooled prevalence of the comorbidities in patients with erosive pustular dermatosis of the scalp [file Image7.jpeg]

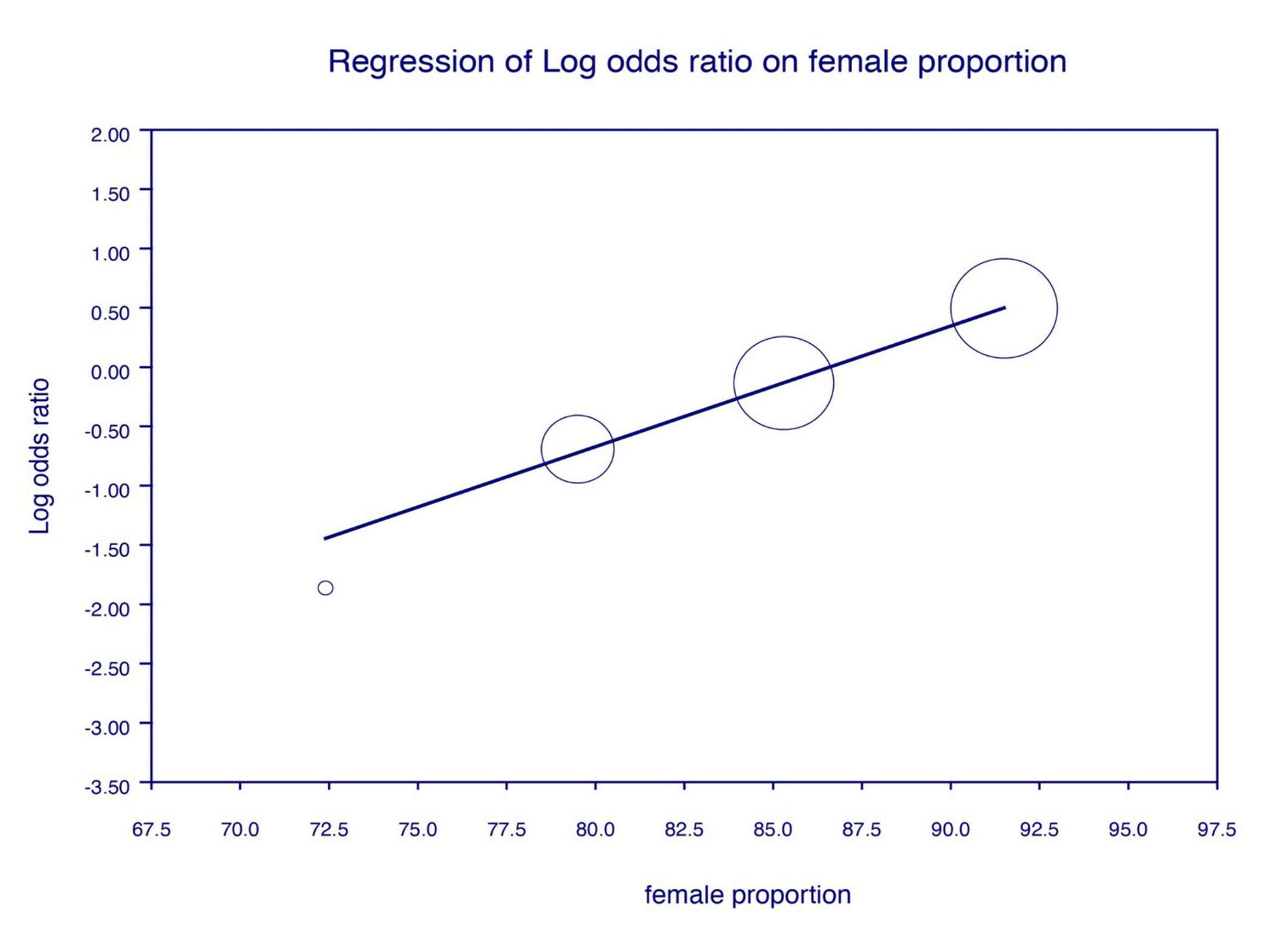

Supplement: Supplementary Figure 8 — Bubble plot for the meta-regression analysis of the pooled odds ratio of depression in patients with lichen planopilaris, using the female proportion as a covariate. [file Image8.jpeg]
