# Supplementary material for: Comorbidities in primary cicatricial alopecia: a systematic review and meta-analysis
Source: Front Immunol. 2025 Aug 29;16:1516407. doi: 10.3389/fimmu.2025.1516407 (PMC12426186; doi:10.3389/fimmu.2025.1516407)
Supplement: Supplementary file 9 [file Table1.docx]

**Supplementary Table 1.** Search strategy

|  | Search strategy |
| --- | --- |
| PubMed  (N = 2363) | 1. "primary scarring alopecia" OR "primary cicatricial alopecia" OR "Lichen planopilaris" OR "Frontal fibrosing alopecia" OR "Graham Little syndrome" OR "Fibrosing alopecia in a pattern distribution" OR "pseudopelade of Brocq" OR "Central centrifugal cicatricial alopecia" OR "Alopecia mucinosa" OR "Keratosis follicularis spinulosa decalvans" OR "Folliculitis decalvans" OR "Dissecting cellulitis" OR "Acne keloidalis" OR "Erosive pustular dermatosis" 2. “discoid lupus erythematosus” AND scalp 3. #1 OR #2 |
| Embase  (N = 3592) | 1. 'primary scarring alopecia' OR 'primary cicatricial alopecia' OR 'lichen planopilaris' OR 'frontal fibrosing alopecia' OR 'graham little syndrome' OR 'fibrosing alopecia in a pattern distribution' OR 'pseudopelade of brocq' OR 'central centrifugal cicatricial alopecia' OR 'alopecia mucinosa' OR 'keratosis follicularis spinulosa decalvans' OR 'folliculitis decalvans' OR 'dissecting cellulitis' OR 'acne keloidalis' OR 'erosive pustular dermatosis' 2. 'discoid lupus erythematosus' AND scalp 3. #1 OR #2 |
| Scopus  (N = 3205) | 1. TITLE-ABS-KEY ( "primary scarring alopecia" OR "primary cicatricial alopecia" OR "Lichen planopilaris" OR "Frontal fibrosing alopecia" OR "Graham Little syndrome" OR "Fibrosing alopecia in a pattern distribution" OR "pseudopelade of Brocq" OR "Central centrifugal cicatricial alopecia" OR "Alopecia mucinosa" OR "Keratosis follicularis spinulosa decalvans" OR "Folliculitis decalvans" OR "Dissecting cellulitis" OR "Acne keloidalis" OR "Erosive pustular dermatosis") 2. TITLE-ABS-KEY (“discoid lupus erythematosus” AND scalp) 3. #1 OR #2 |
