# Supplementary material for: Comorbidities in primary cicatricial alopecia: a systematic review and meta-analysis
Source: Front Immunol. 2025 Aug 29;16:1516407. doi: 10.3389/fimmu.2025.1516407 (PMC12426186; doi:10.3389/fimmu.2025.1516407)
Supplement: Supplementary file 10 [file Table2.docx]

**Supplementary Table 2.** Characteristics and main findings of case-control studies that included patients with lichen planopilaris or frontal fibrosing alopecia

| **Study** | **Study source and design** | **Study population, N** | **Mean age, y/ female, %** | **Control, N** | **Mean age, y/ female, %** | **Findings (number of [case/ control] with a certain comorbidity)** |
| --- | --- | --- | --- | --- | --- | --- |
| Yu, 2024(20) | Nationwide, population-based, cross-sectional study, National Health Insurance Service Database of Korea | 927 LLP/FFA, | 38.0*/ 35.6* | 47,530–47,837 age- and gender-matched normal controls | 38.7*/ 46.8* | Dyslipidemia [198/ 8118], diabetes mellitus [77/ 3711], hypertension [466/ 24454], hyperthyroidism [12/ 385], hypothyroidism [53/ 955] |
|  |  | 157 PPB | 38.0*/ 35.6* | 47,530–47,837 age- and gender-matched normal controls | 38.7*/ 46.8* | Dyslipidemia [21/ 8118], diabetes mellitus [11/ 3711], hypertension [91/ 24454], hyperthyroidism [3/ 385], hypothyroid [7/ 955] |
| Sung, 2023(17) | Nationwide, population-based, cross-sectional study, National Health Insurance Service Database of Korea | 2025 LPP | 47.8/ 58.1 | 40,520 age-, gender-, insurance type- and income-level-matched controls | 47.8/ 58.1 | Systemic lupus erythematosus [34/ 223], Sjogren’s syndrome [21/ 347], Behcet disease [8/ 147], ankylosing spondylitis [61/ 1140], psoriasis [421/ 1900], rheumatoid arthritis [352/ 5336], Crohn’s disease [8/ 219], ulcerative colitis [34/ 344], multiple sclerosis [4/ 22], lichen planus [119/ 461], atopic dermatitis [789/ 6441], allergic rhinitis [1747/ 33142], asthma [790/ 14930], diabetes mellitus [490/ 8536], hyperthyroidism [187/ 3236], hypothyroidism [256/ 4168], thyroiditis (unspecified) [117/ 1953], hypertension [637/ 11509], dyslipidemia [930/ 16567], myocardial infarction [41/ 801], chronic heart failure [82/ 1434], stroke [139/ 2352], transient ischemic attack [96/ 1606], peripheral artery disease [370/ 6833], mood disorders [446/ 7790], anxiety [878/ 16495], non-melanocytic skin cancer [4/ 57], melanoma [2/ 14], vitamin D deficiency [54/ 724], iron deficiency anemia [321/ 5577] |
| Joshi, 2023(13) | Nested-case–control study, National Institute of Health’s All of Us database | 142 LPP | 62.4/ 91.5 | 568 age-, gender-, and race-matched controls | 62.4/ 91.5 | Anxiety [53/ 144], atopic dermatitis [18/ 3], basal cell carcinoma [15/ 13], depression [49/ 138], heart failure [9/ 126], Human Immunodeficiency Virus infection [0/ 4], hypertension [77/ 224], dyslipidemia [74/ 191], hypothyroidism [44/ 91], hyperthyroidism [1/ 0], ischemic heart disease [10/ 14], inflammatory bowel disease [14/ 15], insomnia [30/ 51], melanoma [6/ 4], obesity [39/ 137], psoriasis [11/ 1], rheumatoid arthritis [9/ 11], rosacea [23/ 22], squamous cell carcinoma [5/ 5], systemic lupus erythematosus [9/ 11], type 1 diabetes mellitus [2/ 8], diabetes mellitus [27/ 91], vitiligo [2/ 2] |
| Porriño-Bustamante, 2022(40) | Cross-sectional, case-control study | 101 FFA | 63.5/ 100 | 40 age- and gender-matched controls | 63.1/ 100 | Lentigines [69, 19], actinic keratosis [17, 3], basal cell carcinoma [10, 3], squamous cell carcinoma [0, 0] |
| Nasimi, 2022(15) | Retrospective, case-control study | 208 LPP | 47.2/ 76.9 | 208 age-, gender-, and BMI-matched controls | 42.4/ 66.8 | Thyroid disease [36, 28], hypothyroidism [33, 27], thyroid goiter/nodules/cancer [3, 1], cardiovascular disorders [11, 22], hypertension [26, 35], dyslipidemia [87, 36] |
| Bazotti, 2022(34) | Case-control study | 30 FFA | 64.5/ 100 | 30 age-, gender-, and ethnicity-matched control | 64.2/ 100 | Hypothyroidism [7, 3], systemic lupus erythematosus [2, 1] |
| Arasu, 2022(33) | Case-control study | 100 FFA | 63/ 100 | 100 gender-matched controls with female pattern hair loss | 51/ 100 | Vitamin D deficiency [4, 2] |
| Trager, 2021(19) | Cross-sectional study | 204 LPP | NP/ 72.4 | 1,189,507 unmatched controls without FFA/LPP | NP/ 56.9 | Atopic dermatitis/ allergic rhinitis/ asthma [6, 61733], Graves disease [0, 1668], hypothyroidism [9, 39620], Hashimoto thyroiditis [1, 3406], hyperthyroidism [0, 3573], Celiac disease [1, 3538], multiple sclerosis [1, 6894], psoriasis [6, 4545], inflammatory bowel disease [5, 20287], systemic lupus erythematosus [1, 3002], rheumatoid arthritis [4, 11710], Sjogren syndrome [0, 876], vitiligo [0, 1085] , type 1 diabetes mellitus [1, 5243], diabetes mellitus [3, 61354], lichen planus [4, 318] |
|  |  | 174 FFA | NP/ 91.9 | 1,189,507 unmatched controls without FFA/LPP | NP/ 56.9 | Atopic dermatitis/ allergic rhinitis/ asthma [8, 61733], Graves disease [0, 1668], hypothyroidism [12, 39620], Hashimoto thyroiditis [1, 3406], hyperthyroidism [1, 3573], Celiac disease [1, 3538], multiple sclerosis [1, 6894], psoriasis [8, 4545], inflammatory bowel disease [3, 20287], systemic lupus erythematosus [3, 3002], rheumatoid arthritis [3, 11710], Sjogren syndrome [0, 876], vitiligo [2, 1085], type 1 diabetes mellitus [0, 5243], diabetes mellitus [7, 61354], lichen planus [1, 318] |
| Ramos, 2021(41) | Multicenter, prospective, case-control study | 451 FFA | 53/ 96 | 451 gender-matched controls with nonscarring alopecia | 49/ 96 | Allergic rhinitis [132, 147], gastritis/reflux [106, 92], sinusitis [116, 120], dyslipidemia [118, 100], thyroid disorder [107, 70], depression [92, 76], hypertension [91, 95], rosacea [60, 31], asthma [55, 65], urticaria [43, 36], panic syndrome [39, 29], diabetes mellitus [28, 32], rheumatoid arthritis [27, 21], psoriasis [22, 18], malignancies [14, 4], vitiligo [9, 4], lupus [9, 4] |
| Leecharoen, 2021(37) | Case-control study | 50 FFA | 60.2/ 100 | 100 age- and gender-matched controls | 60.2/ 100 | Thyroid disease [4, 5], vitiligo [2, 0], diabetes mellitus [10, 10], hypertension [34, 30], dyslipidemia [26, 34], seborrhea [6, 14], hirsutism [0, 0], acne [2, 1], obesity [36, 35], infertility [8, 4] |
| Conic, 2021 (10) | Population-based, cross-sectional study | 3170 LPP | NP/ 89 | 63,442,000 unmatched controls | NP/ 54 | Hypertension [1480, 11555240], obesity [850, 4154570], dyslipidemia [1660, 9265060], diabetes mellitus [550, 4728620], metabolic syndrome [90, 182810], coronary artery disease [240, 2997630], atrial fibrillation [140, 1813160], myocardial infarction [100, 1338570] |
| Rudnicka, 2020(42) | Prospective, multi-center, case-control study | 20 FFA | 39.7/ 95 | 24 gender-matched controls | 41.2/ NP | Allergic contact dermatitis [13, 9] |
| Manatis-Lornell, 2020(14) | Retrospective, case-control study | 232 LPP | 61.7/ 85 | 194 age-, gender-, and race/ethnicity-matched controls | 65/ 85.1 | Rosacea [56, 13], cardiovascular disease [103, 90], coronary artery disease [19, 14], hypertension [94, 78], chronic heart failure [6,17], arrhythmia [16, 16], peripheral artery disease [3, 5], stroke [5, 17], congenital heart disease [1, 0], other cardiovascular disease [5, 0], autoimmune disease not otherwise specified [24, 23], rheumatoid arthritis [1, 4], lupus [8, 2], Celiac disease [3, 1], Sjogren syndrome [3, 1], polymyalgia rheumatica [2, 1], multiple sclerosis [1, 1], ankylosing spondylitis [0, 0], type 1 diabetes mellitus [1, 3], alopecia areata [7, 0], vasculitis [0, 2], temporal arteritis [0, 0], glaucoma [0, 11], other autoimmune diseases [0, 1], thyroid diseases not otherwise specified [66, 46], thyroid nodules [23, 22], hypothyroidism [48, 27], hyperthyroidism [4, 4], goiter [10, 13], thyroiditis (unspecified) [11, 8], thyroid cancer [3, 2], other thyroid disorders [1, 0], depression [63, 58], anxiety [79, 62], dyslipidemia [97, 81], polycystic ovary syndrome [5, 1], leiomyoma [44, 29], melanoma/squamous cell carcinoma/basal cell carcinoma [53, 20] |
| Donati, 2020(36) | Cross-sectional study | 24 FFA | 48.6/ 100 | 468 unmatched controls | 38/ 94.4 | Autoimmune disease [8, 55], thyroiditis (unspecified) [6, 35], lupus [0, 3], other autoimmune diseases [3, 21], allergy [17, 211], allergic contact dermatitis [5, 35] |
| Porriño-Bustamante, 2019(39) | Case-control study | 99 FFA | 63.3/ 100 | 40 age- and gender-matched controls | 61.7/ 100 | Hypertension [45, 16], diabetes mellitus [14, 5], dyslipidemia [44, 17], rosacea [61, 12] |
| Moreno-Arrones, 2019(38) | Multi-center, prospective, case-control study | 308 FFA | 60/ 93.4 | 347 age- and gender-matched controls | 58.4/ 79.9 | Type 1 diabetes mellitus [1, 4], rheumatoid arthritis [21, 12], lupus [1, 0], vitiligo [7, 2], lichen planus pigmentosus [9, 2], rosacea [38, 20], Dupuytren disease [1, 0], visceral/peritoneal fibrosis [1, 3], arthrofibrosis [2, 3], hypothyroidism [60, 39], hyperthyroidism [1, 3], keloid [7, 3], breast cancer [10, 13], ovarian cancer [0, 2] |
| Fertig, 2018(12) | Retrospective, case-control study | 87 LPP | 56/ 72.4 | 323 age-, gender-, ethnicity-, and race-matched controls with psoriasis or actinic keratosis | 50.9/ 83.3 | Hypertension [4, 17], diabetes mellitus [1, 8], dyslipidemia [6, 13], heart disease [2, 12], hepatitis C virus infection [0, 1], rheumatoid arthritis [0, 0], systemic lupus erythematosus [3, 1], vitiligo [4, 5], alopecia areata [5, 17], psoriasis [2, 11], Crohn’s disease [0, 1], multiple sclerosis [0, 4], bone, connective tissue, skin, and breast cancer [5, 25], other and unspecified cancer [1, 6], lymphatic and hematopoietic tissue cancer [2, 4], respiratory and intrathoracic organs cancer [0, 2], genitourinary organs cancer [0, 3], depression [0, 11], hyperthyroidism [0, 4], hypothyroid [0, 20], fatty liver [0, 4], chronic airway obstruction [0, 2], sleep apnea [0, 5], allergic rhinitis [3, 6] |
|  |  | 119 FFA | 59.5/ 100 | 323 age-, gender-, ethnicity-, and race-matched controls with psoriasis or actinic keratosis | 50.9/ 83.3 | Hypertension [12, 17], diabetes mellitus [4, 8], dyslipidemia [10, 13], heart disease [9, 12], hepatitis C virus infection [1, 1], rheumatoid arthritis [2, 0], systemic lupus erythematosus [6, 1], vitiligo [4, 5], alopecia areata [16, 17], psoriasis [2, 11], Crohn disease [0, 1], multiple sclerosis [1, 4], bone, connective tissue, skin, and breast cancer [9, 25], other and unspecified cancer [1, 6], lymphatic and hematopoietic tissue cancer [0, 4], respiratory and intrathoracic organs cancer [0, 2], genitourinary organs cancer [0, 3], depression [3, 11], hyperthyroidism [2, 4], hypothyroidism [8, 20], fatty liver [2, 4], chronic airway obstruction [0, 2], sleep apnea [1, 5], allergic rhinitis [2, 6] |
| Conic, 2018(11) | Case-control study | 187 LPP | 57/ 91.4 | 56 age-, gender-, race-, and BMI-matched controls with seborrheic dermatitis | 54/ 89.3 | Dyslipidemia [35, 15], hypertension [46, 20] |
| Buendía-Castaño, 2018(35) | Case-control study | 104 FFA | 62.6/ 100 | 208 age- and gender-matched controls | 62.4/ 100 | Breast cancer [8, 5], polycystic ovarian syndrome [9, 15] |
| Brankov, 2018(9) | Retrospective, case-control study | 334 LPP | 52.2/ 79.5 | 78 age- and race-matched controls with seborrheic dermatitis without hair loss | 54.8/ 93.1 | Allergic rhinitis [50, 19], atopic dermatitis [42, 10], asthma [33, 12], Hashimoto’s thyroiditis [21, 0], systemic lupus erythematosus [5, 1], rheumatoid arthritis [4, 1], psoriasis [7, 1], sarcoidosis [4, 1], celiac disease [4, 2], ulcerative colitis [4, 2], vitiligo [2, 1], Sjogren’s syndrome [2, 1], limited scleroderma and systemic sclerosis [3, 0], hypothyroidism [81, 10], other thyroid disease [25, 6], hyperthyroidism [4, 1], goiter [10, 3], thyroid nodules [3, 1], subacute thyroiditis [1, 0], diabetes mellitus [39, 17], dyslipidemia [129, 41], obesity [109, 27], hirsutism [38, 1], hyperparathyroidism [3, 3], vitamin D deficiency [167, 51], anemia [60, 17], iron deficiency [27, 5], anxiety [35, 10], depression [52, 21], sleep problems [25, 23] |
| Nguyen, 2016(16) | Case-control study | 28 LPP | NP/ NP | 31 age- and gender-matched controls | NP/ NP | Thyroid disease [6, 5] |
|  |  | 35 FFA | NP/ NP | 31 age- and gender-matched controls | NP/ NP | Thyroid disease [7, 5] |
| Aldoori, 2016(32) | Case-control study | 105 FFA | 63.8/ 100 | 100 age- and gender-matched controls | 59.4/ 100 | Thyroid disease [20, 7] |
| Toossi, 2015(18) | Case-control study | 26 LPP | 52.5/ 69.2 | 30 age- and gender-matched controls | 54.5/ 70 | Thyroid disease [11, 2], hypothyroidism [7, 0] |
| Atanaskova Mesinkovska, 2014(8) | Retrospective, case-control study | 166 LPP | 55.1/ 88 | 81 age- and gender-matched controls with seborrheic dermatitis without alopecia | 52.7/ 79 | diabetes mellitus [15, 15], dyslipidemia [42, 37], thyroid disease [57, 9], hypothyroidism [48, 7], Hashimoto thyroiditis [10, 0], hyperthyroidism [2, 0], goiter [3, 0], thyroid nodules [2, 1], thyroid tumor [1, 0], chronic lymphocytic thyroiditis [1, 0] |

* Data from the whole cohort

FFA, frontal fibrosing alopecia; LPP, lichen planopilaris; PPB, pseudopelade of Brocq
