# Supplementary material for: Comorbidities in primary cicatricial alopecia: a systematic review and meta-analysis
Source: Front Immunol. 2025 Aug 29;16:1516407. doi: 10.3389/fimmu.2025.1516407 (PMC12426186; doi:10.3389/fimmu.2025.1516407)
Supplement: Supplementary file 11 [file Table3.docx]

**Supplementary Table 3.** Characteristics and main findings of case-control studies that included patients with central centrifugal cicatricial alopecia

| **Study** | **Study source and design** | **Study population, N** | **Mean age, y/ female, %** | **Control, N** | **Mean age, y/ female, %** | **Comorbidity [case, control]** |
| --- | --- | --- | --- | --- | --- | --- |
| Joshi, 2024(86) | Nested-case–control study, National Institute of Health’s All of Us database | 201 CCCA | 55.4/ 95.5 | 201 age-, gender-, race-, ethnicity-matched controls | 55.4/ 95.5 | Atopic dermatitis [14/ 3], allergic rhinitis [83/ 21], anxiety [105/ 34], asthma [61/ 22], autoimmune conditions [51/ 13], depression [97/ 45], hypertension [122/ 46], dyslipidemia [161/ 64], diabetes mellitus [94/ 27] |
| Leung, 2023(88) | Population-based, case-control study | 53 CCCA | 51.3/ 100 | 212 age- and gender-matched controls | 50.3/ 100 | Dyslipidemia [35/ 35], hypertension [37/ 123], obesity [34/ 120], diabetes mellitus [13/ 43], allergic rhinitis [6/ 0], anxiety [6/ 0], vitamin D deficiency [5/ 0], malignancies [5/ 0], gastroesophageal reflux disease [4/ 0], asthma [4/ 0], sleep disorders [3/ 0], hypothyroidism [3/ 0], anemia [3/ 0], hirsutism [2/ 0], seborrheic dermatitis [1/ 0], acne [1/ 0], iron deficiency [1/ 0], systemic lupus erythematosus [1/ 0], vitiligo [1/ 0] |
| Jafari, 2023(85) | Retrospective, case-control study | 153 CCCA | 57.2/ 100 | 153 age-, gender-, ethnicity-, and insurance status-matched nonscarring alopecia controls | 56.8/ 100 | Hypertension [129, 126], obesity [44, 44], peripheral artery disease [6, 5], dyslipidemia [74, 71], diabetes mellitus [77, 72], hyperparathyroidism [6, 6], adrenal nodule [4, 3], hidradenitis suppurativa [4, 4], human immunodeficiency virus infection [7, 8], systemic lupus erythematosus [10, 4], discoid lupus erythematosus [1, 3], sarcoidosis [6, 2], Crohn disease [2, 0], vitiligo [2, 2], Sjogren syndrome [3, 0], Hashimoto thyroiditis [8, 8], Graves disease [3, 6], endometrial hyperplasia [4, 0], leiomyoma [8, 6], hirsutism [5, 1], bipolar disorder [3, 0], depression [18, 17], anxiety [9, 6], schizophrenia [4, 0], seborrheic dermatitis [30, 28], bacterial scalp infection [12, 9], fungal scalp infection [13, 11] |
| Roche, 2022(91) | Retrospective, case-control study | 395 CCCA | 57/ 100 | 39,280 age-, gender-, and ethnicity-matched control | 55/ 100 | Diabetes mellitus [105, 7002], obesity [356, 38405] |
| Samrao, 2021(92) | Retrospective case-control cohort study, Kaiser Permanente Northern California members | 427 CCCA | 50.1/ 100 | 1,281 age-, gender-, and ethnicity-matched controls | NP/ 100 | Obesity [375, 1063], diabetes mellitus [79, 268], hypertension [240, 751], lung disease [0, 12], atherosclerosis [89, 252], cirrhosis [0, 1], nonalcoholic steatohepatitis [8, 33], end-stage renal disease [5, 15], leiomyoma [121, 313] |
| McKenzie, 2021(89) | Cross-sectional study | 270 CCCA | NP/ 100 | 153 unmatched controls with alopecia areata or psoriasis | NP/ NP | Depression/anxiety [27,17] |
| Brown-Korsah, 2021(99) | Cross-sectional study | 742 CCCA | NP/ 100 | 224,674 age-, gender-, and race-matched controls | NP/ 100 | Breast cancer [35, 4079], colorectal cancer [1, 701] |
| Narasimman, 2020(90) | Retrospective, case-control study | 74 CCCA | 46.9/ 100 | 96 age-, gender-, and race-matched controls with other hair loss conditions | 47.4/ 100 | Diabetes mellitus [17, 21], hypertension [45, 54], leiomyoma [14, 7] |
| Dina, 2018(84) | Case-control study | 447 CCCA | NP/ 100 | 486,657 age-, gender-, and race-matched controls | NP/ 100 | Leiomyoma [62, 16212] |
| Kyei, 2011(87) | Population-based, cross-sectional study | 52 CCCA | 58/ 100 | 224 gender-matched controls without CCCA | 40/ 100 | Diabetes mellitus [9, 13], thyroid disease [4, 20], bacterial skin infection [10, 22], tinea corporis [4, 25], tinea capitis [7, 23], vaginal yeast infection [21, 112], acne [38, 154], hirsutism [22, 117], scar formation/keloid [2, 9], seborrheic dermatitis [14, 55], atopic dermatitis [2, 35], contact dermatitis [6, 18] |

CCCA, central centrifugal cicatricial alopecia
