# Supplementary material for: Comorbidities in primary cicatricial alopecia: a systematic review and meta-analysis
Source: Front Immunol. 2025 Aug 29;16:1516407. doi: 10.3389/fimmu.2025.1516407 (PMC12426186; doi:10.3389/fimmu.2025.1516407)
Supplement: Supplementary file 12 [file Table4.docx]

**Supplementary Table 4.** Characteristics and main findings of case-control studies that included patients with acne keloidalis nuchae, dissecting cellulitis or folliculitis decalvan

| **Study** | **Study source and design** | **Study population, N** | **Mean age, y/ female, %** | **Control, N** | **Mean age, y/ female, %** | **Comorbidity [case, control]** |
| --- | --- | --- | --- | --- | --- | --- |
| Yu, 2024(20) | Nationwide, population-based, cross-sectional study, National Health Insurance Service Database of Korea | 5651 FD | 38.0*/ 35.6* | 47,530–47,837 age- and gender-matched normal controls | 38.7/ 46.8 | Dyslipidemia [1088, 8118], diabetes mellitus [515, 3711], hypertension [3081, 24454], hyperthyroidism [51, 385], hypothyroidism [118, 955] |
|  |  | 4470 DC | 38.0*/ 35.6* |  | 38.7/ 46.8 | Dyslipidemia [930, 8118], diabetes mellitus [506, 3711], hypertension [2374, 24454], hyperthyroidism [27, 385], hypothyroidism [56, 955] |
| Tran, 2022(105) | Retrospective, case-control study | 33 DC | 39.6/ 12.1 | 304 ethnicity-matched keloid patients | 44.4/ 66.4 | Overweight [7, 71], obesity [16, 169] |
| Kridin, 2021(109) | Retrospective, population-based, cross-sectional study, Clalit Health Services database | 2677 AKN | 34.5/ 4.3 | 13,190 age-, gender-, and ethnicity-matched controls | 34.5/ 4.3 | Hidradenitis suppurativa [28, 39] |
| Saka, 2020(118) | Multi-center, case-control study | 101 AKN | 34.9/ 100 | 202 age-matched controls | 35.6/ NP | Obesity [65, 55], systolic hypertension [24, 16], diastolic hypertension [34, 59] |
| Kridin, 2020(110) | Retrospective, population-based, cross-sectional study, Clalit Health Services database | 2677 AKN | 34.5/ 4.3 | 13,190 age-, gender-, and ethnicity-matched controls | 34.5/ 4.3 | Obesity [1055, 2349], hypertension [460, 1348], dyslipidemia [881, 3098], diabetes mellitus [497, 1115], metabolic syndrome [431, 870] |

AKN, acne keloidalis nuchae; DC, dissecting cellulitis; FD, folliculitis decalvan
