# Supplementary material for: Comorbidities in primary cicatricial alopecia: a systematic review and meta-analysis
Source: Front Immunol. 2025 Aug 29;16:1516407. doi: 10.3389/fimmu.2025.1516407 (PMC12426186; doi:10.3389/fimmu.2025.1516407)
Supplement: Supplementary file 13 [file Table5.docx]

**Supplementary** **Table 5.** Characteristics and main findings of descriptive studies

| **Study** | **Study source and design** | **Study population, N** | **Mean age, y/** | **Female, %** | **Comorbidity, N** |
| --- | --- | --- | --- | --- | --- |
| Sarkis, 2024(103) | Retrospective, multicenter, descriptive study | 150 FD | 38.9 | 100 | Polycystic ovarian syndrome 11, androgenetic alopecia 13, autoimmune alopecia 9 |
| Lyakhovitsky, 2024(29) | Retrospective, cohort study | 126 LPP | 47.9 | 70.6 | Lichen planus 17, seborrheic dermatitis 21, vitiligo 2, atopic dermatitis 4, psoriasis 1, rosacea 1, diabetes mellitus 7, hypertension 15, dyslipidemia 28, obesity 13, lupus 1, scleroderma 1, Sjogren syndrome 2, Raynaud phenomenon 2, fibromyalgia 2, gout 1, allergic rhinitis 8, food allergy 1, allergic conjunctivitis 1, asthma 1, chronic obstructive pulmonary disease 1, ischemic heart disease 1, arrhythmia 2, Celiac disease 1, gastroesophageal reflux disease 4, peptic ulcer 2, irritable bowel syndrome 1, colonic polyp 1, diverticulitis 1, benign prostatic hyperplasia 1, hypothyroidism 15, Hashimoto thyroiditis 2, thyroid nodules 3, migraine 2, cerebrovascular disease 1, epilepsy 2, meningioma 1, insomnia 14, anxiety 12, depression 11, post-traumatic stress disorder 3, schizoaffective disorder 1, basal cell carcinoma 3, melanoma 1, breast cancer 3, cervical cancer 1, lung cancer 1, squamous cell carcinoma of the vocal cord 1, thyroid cancer 1, autoimmune hemolytic anemia 1, IgA vasculitis 1 |
| Lobato-Berezo, 2024(113) | Retrospective, multicenter, descriptive study | 79 AKN | 35 | 5.1 | Obesity 9, hypertension 5, dyslipidemia 10, diabetes mellitus 4, hypothyroidism 0, acanthosis nigricans 7, keloid 9, pseudofolliculitis barbae 15, hidradenitis suppurativa 9, acne conglobata 11, sinus pilonidal 1, DC 5, FD 16 |
| Carrascoza, 2024(23) | Retrospective, descriptive study | 103 LPP | NP | 66 | Hashimoto thyroiditis 17, hyperparathyroidism 1, vitiligo 0, psoriasis 0, alopecia areata 0, urticaria 0, scleroderma 2, systemic lupus erythematosus 2, dermatomyositis 2, hypertension 6, dyslipidemia 3, diabetes mellitus 5, stroke 2, myocardial infarction 1, keratosis pilaris 1, rosacea 0, androgenetic alopecia 14, lichen planus pigmentosus 1, mucosal lichen planus 8, cutaneous lichen planus 4, nail lichen planus 1, allergic rhinitis 1, anxiety 1, depression 1, epilepsy 1 |
|  |  | 138 FFA | NP | 96.4 | Hashimoto thyroiditis 35, hyperparathyroidism 0, vitiligo 4, psoriasis 3, alopecia areata 2, urticaria 4, scleroderma 2, systemic lupus erythematosus 6, dermatomyositis 0, hypertension 9, dyslipidemia 10, diabetes mellitus 4, menopause 5, stroke 0, myocardial infarction 0, keratosis pilaris 1, rosacea 8, androgenetic alopecia 12, lichen planus pigmentosus 22, mucosal lichen planus 3, cutaneous lichen planus 2, nail lichen planus 1, allergic rhinitis 1, anxiety 1, depression 1, epilepsy 0 |
| Xavier de Brito, 2023(82) | Retrospective, multicenter, descriptive study | 104 FFA with lichen planus pigmentosus | 60.5 | 97.1 | Obesity 19, thyroid disease 21, other type of lichen 4, hepatitis C virus infection 4 |
| Verma, 2023(81) | Retrospective, descriptive study | 21 FFA | 48.3 | 90.5 | Lichen planus pigmentosus 6, androgenetic alopecia 2, lichen planopilaris 2 |
| Shamloul, 2023(122) | Retrospective, descriptive study | 80 EPD | NP | 33.7 | Squamous cell carcinoma 34, basal cell carcinoma 7, atypical fibroxanthoma 2, pleomorphic dermal sarcoma 1 |
| Saceda-Corralo, 2023(71) | Cross-sectional, descriptive study | 223 FFA | 61 | 100 | Hashimoto thyroiditis 11, Graves disease 3, Sjogren syndrome 1, pernicious anemia 1, ankylosing spondylitis 1, DLE 1, rosacea 28, breast cancer 8 |
| Parker, 2023(115) | Prospective, descriptive study | 28 AKN | 32.8 | 0 | Pseudofolliculitis barbae 15, acanthosis nigricans 7, acne conglobata 4 |
| Oulad Ali, 2023(65) | Prospective, descriptive study | 38 FFA | 53 | 100 | Lichen planus pigmentosus 14, rosacea 11, vitiligo 1, psoriasis 1, acne 1, thyroid disease 9, dyslipidemia 8, diabetes mellitus 3, hypertension 7, coronary artery disease 1, atopy 2, Celiac disease 1, psoriatic arthritis 1, epilepsy 1 |
| Onamusi, 2023(96) | Retrospective, descriptive study | 100 CCCA | 49.9 | 100 | Seborrheic dermatitis 39, anemia 30, thyroid 16, diabetes mellitus 13 |
| Lyakhovitsky, 2023(101) | Retrospective, cohort study | 192 FD | 28.5 | 8.3 | Atopic dermatitis 7, psoriasis 4, lichen planus 1, alopecia areata 2, acne 70, hidradenitis suppurativa 17, pilonidal sinus 8 |
| Jackson, 2023(95) | Retrospective, descriptive study | 108 CCCA | 43 | 0 | Diabetes mellitus 0, latent tuberculosis 3 |
| Gharaei Nejad, 2023(26) | Cross-sectional, descriptive study | 60 LPP | 43.6 | 73.3 | Vitiligo 3, asthma/allergy 11, hypothyroidism 13, diabetes mellitus 7, hypertension 10, anemia 12, vitamin D deficiency 18, obesity 8 |
| García, 2023(50) | Retrospective, descriptive study | 118 FFA | 64 | 97.5 | Hypertension 31, dyslipidemia 24, hypothyroidism 21, arthrosis 11, anxiety 10, diabetes mellitus 8, osteoporosis 8 |
| Carmona-Rodríguez, 2023(46) | Retrospective, descriptive study | 306 FFA | 59.5 | 96.7 | Lichen planopilaris 30, cutaneous lichen planus 5, oral lichen planus 10, genital lichen planus 3, hypothyroidism 70, alopecia areata 12, psoriasis 8, vitiligo 5 |
| Balazic, 2023(94) | Retrospective, descriptive study | 185 CCCA | 53.7 | 100 | Diabetes mellitus 44, dyslipidemia 65, hyperthyroidism 1, hypothyroidism 6, other thyroid diseases 4, hypertension 95 |
| Umar, 2022(117) | Retrospective, descriptive study | 108 AKN | 35.8 | 0 | Cutis verticis gyrata 4 |
| Starace, 2022(75) | Cross-sectional, multicenter, descriptive study | 188 FFA | 62 | 98.4 | Androgenetic alopecia 73, lichen planopilaris 35, lichen planus 10 |
| Salas-Callo, 2022(73) | Prospective, descriptive study | 50 FFA | 57.7 | 88 | Lichen planus pigmentosus 14, rosacea 0 |
| Rocha, 2022(69) | Retrospective, descriptive study | 27 FFA | 63.9 | 96.3 | Lichen planus pigmentosus 6, hypertension 18, diabetes mellitus 8, dyslipidemia 8, hypothyroidism 7 |
| Moussa, 2022(64) | Retrospective, descriptive study | 13 FFA | 46.1 | 0 | Rosacea 4, cutaneous lichen planus 0, mucosal lichen planus 0, asthma 1, atopic dermatitis 1, contact dermatitis 1, dyslipidemia 1, hypertension 1, ulcerative colitis 1, androgenetic alopecia 3 |
| Melo, 2022(108) | Retrospective, multicenter, descriptive study | 17 DC | 17 | 100 | Hidradenitis suppurativa 7, acne conglobata 4, polycystic ovarian syndrome 1, androgenetic alopecia 1, congenital triangular alopecia 1 |
| Lobato-Berezo, 2022(59) | Retrospective, multicenter, descriptive study | 39 FFA | 69 | 0 | Androgenetic alopecia 30, hypothyroidism 2, benign prostatic hyperplasia 13, prostate cancer 6, rosacea 12 |
| Jiang, 2022(56) | Retrospective, descriptive study | 169 DC | 32 | 0 | Diabetes mellitus 13, eczema 13, seborrheic dermatitis 18, hypertension 9, fatty liver 6, gout 6, acanthosis nigricans 2, urticaria 2, psoriasis 2, ankylosing spondylitis 1 |
| Dorgham, 2022(49) | Retrospective, descriptive study | 58 FFA | 50.5 | 100 | Rosacea 2, lichen planus 2, thyroid dysfunction 9, vitiligo 1, melasma 2, telogen eflluvium 18, LPP 11, androgenetic alopecia 6, alopecia areata 2 |
| Doche, 2022(25) | Cross-sectional, descriptive study | 10 LPP | 58.8 | 89.2 | Lichen planus pigmentosus 0, nail lichen planus 2, oral lichen planus 1, cutaneous lichen planus 1 |
|  |  | 27 FFA | 59.7 | 92.6 | Lichen planus pigmentosus 6, nail lichen planus 0, oral lichen planus 1, cutaneous lichen planus 0 |
| Doche, 2022(48) | Retrospective, multicenter, descriptive study | 33 FFA | 53.1 | 0 | Obesity 2, hypertension 8, anxiety/depression 4, hypothyroidism 3, androgenetic alopecia 21, rosacea 11, LPP 3, cutaneous lichen planus 2, lichen planus pigmentosus 1, nail lichen planus 0, oral lichen planus 0 |
| Collins, 2022(47) | Retrospective, descriptive study | 27 CCCA | NP | NP | Vitamin D deficiency 25 |
| Ali, 2022(93) | Retrospective, descriptive study | 35 CCCA | 49.2 | 91.4 | Diabetes mellitus 20 |
| Uzunçakmak, 2021(79) | Retrospective, descriptive study | 16 FFA | 51.9 | 100 | Hashimoto thyroiditis 3, hypertension 3, ankylosing spondylitis 1, hyperthyroidism 1, psoriasis 1, mixed connective tissue disease 1 |
| Umar, 2021(116) | Retrospective, descriptive study | 108 AKN | 35.8 | 0 | Acne 42, pseudofolliculitis barbae 29, FD/ DC 8, hidradenitis suppurativa 0, keloid 2 |
| Rossi, 2021(70) | Prospective, descriptive study | 8 FFA | 58.8 | 0 | Hypertension 3, alopecia areata 1, psoriasis 1, dyslipidemia 1, lichen sclerosus 2, hypothyroidism 0, hyperthyroidism 0 |
| Pindado-Ortega, 2021(68) | Retrospective, descriptive study | 224 FFA | 61.2 | 99.1 | Rosacea 40, hypothyroidism 26 |
| Michelerio, 2021(121) | Retrospective, descriptive study | 50 EPD | 72 | 28 | Hypothyroidism 1, psoriasis 2, prostate cancer 5, colon cancer 1, laryngeal cancer 1, actinic keratosis 11, diabetes mellitus 2, basal cell carcinoma 2, squamous cell carcinoma 4 |
| Melo, 2021(62) | Retrospective, multicenter, descriptive study | 38 FFA | 55 | 97.4 | Lichen planus pigmentosus 2, autoimmune disease 4 |
| Jerjen, 2021(55) | Retrospective, descriptive study | 24 FAPD | 60.7 | 100 | Alopecia areata 3, celiac disease 2, pernicious anemia 1, rheumatoid arthritis 2, frontal fibrosing alopecia 2, scalp psoriasis 1, traction alopecia 1 |
| Grassi, 2021(52) | Retrospective, descriptive study | 119 FFA | 66.5 | 93.3 | Hypothyroidism 21, hypertension 19, dyslipidemia 17, androgenetic alopecia 19, alopecia areata 5, lichen planus 19, lichen sclerosus 15 |
| Feng, 2021(107) | Retrospective, descriptive study | 12 DC | 28.4 | 0 | Hidradenitis suppurativa 4, hyperthyroid 1, hypertension 1, hyperuricemia 2, diabetes mellitus 1, myasthenia gravis 1 |
| Conic, 2021(24) | Retrospective, descriptive study | 58 LPP | 56.6 | 94.8 | Vitamin D deficiency 20 |
|  |  | 29 CCCA | 55.2 | 100 | Vitamin D deficiency 16 |
| Cantwell, 2021(22) | Retrospective, descriptive study | 19 LPP | 51.1 | 0 | Hypertension 7, obesity 7, dyslipidemia 6, benign prostatic hyperplasia 5, tubular adenoma 5, mechanical back pain 4, degenerative arthritis 3, erectile dysfunction 3, gastroesophageal reflux disease 3, thyroid disease 3, depression 2, diabetes mellitus 2, nephrolithiasis 2, obstructive sleep apnea 2, prostatitis 2, pulmonary nodule 2, aortic stenosis 1, essential tremor 1, bicuspid aortic valve 1, cataracts 1, cholelithiasis 1, coronary artery disease 1, fasting hyperglycemia 1, focal segmental glomerulosclerosis 1, generalized anxiety disorder 1, gout 1, growth hormone deficiency 1, hepatitis C virus infection 1, hypospadias 1, IgA nephropathy 1, intestinal obstruction 1, atrial fibrillation 1, peripheral arterial disease 1, precocious puberty 1, hyperparathyroidism 1, seronegative polyarthritis 1, Tetralogy of Fallot 1, deep vein thrombosis/ pulmonary embolism 1, androgenetic alopecia 5, basal cell carcinoma 3, dermatitis 3, acne 1, condyloma acuminatum 1, ichthyosis hystrix 1, lichen sclerosus 1, seborrheic dermatitis 1 |
| Adotama, 2021(43) | Retrospective, descriptive study | 140 FFA | 60 | 100 | Androgenetic alopecia 17, alopecia areata 8, CCCA 3, seborrheic dermatitis 5, systemic lupus erythematosus 3, telogen effluvium 6, traction alopecia 0, dyslipidemia 9, hypertension 8, hypo/hyperthyroidism 14 |
| Suchonwanit, 2020(77) | Retrospective, descriptive study | 56 FFA | 51.3 | 96.4 | Androgenetic alopecia 7, seborrheic dermatitis 5, LPP 1, lichen planus pigmentosus 5, lichen planus 1, discoid lupus erythematosus 1, diabetes mellitus 10, hypertension 8, dyslipidemia 7, hyperthyroidism 2 |
| Saceda-Corralo, 2020(72) | Prospective, cohort study | 57 FFA | 62.4 | 100 | Rosacea 24 |
| Panchaprateep, 2020(66) | Retro-prospective, cohort study | 58 FFA | 61 | 100 | Lichen planopilaris 15, androgenetic alopecia 28, lichen planus pigmentosus 14, rosacea 0, fibrosing alopecia in a pattern distribution 9 |
| McSweeney, 2020(61) | Cross-sectional, descriptive study | 711 FFA | 66 | 100 | Oral lichen planus 32, genital lichen planus 22, cutaneous lichen planus 7, nail lichen planus 2, lichen planus 60, autoimmune disease 141, autoimmune thyroid disease 88, celiac disease 10, pernicious anemia 8 |
| Maldonado Cid, 2020(60) | Retrospective, descriptive study | 75 FFA | 61 | 97.3 | Hypothyroidism 11, rosacea 15, androgenetic alopecia 42 |
| Larkin, 2020(28) | Retrospective, descriptive study | 232 LPP | 59.8 | 100 | Depression 106, anxiety 97, sleep problem 68, neuropathy 13, migraines 11, dementia 5, thyroid disease 71, hypothyroidism 54, Hashimoto thyroiditis 21, Graves disease 6, subacute thyroiditis 6, thyroid nodule 4, thyroid cancer 1, adrenal adenoma 2, hypertension 51, arthritis 41, dyslipidemia 36, coronary artery disease 18, obesity 17, varicose veins/ venous stasis 15, arrhythmia 12, asthma 10, chronic obstructive pulmonary disease 4, gastrointestinal autoimmune diseases 34, celiac disease 8, ulcerative colitis 11, Sjogren syndrome 12, Crohn disease 4, type 1 diabetes mellitus 5, gastroesophageal reflux disease 17, colon polyps 15, hepatitis B virus infection 11, pernicious anemia 4, pancreatitis 2, hepatitis C virus infection 2, osteoporosis 28, vitamin D deficiency 22, anemia 11, renal disease 19, cataract/ glaucoma/ macular degeneration 15, rheumatoid arthritis 10, fibromyalgia 8, carpal tunnel syndrome 5, systemic lupus erythematosus 2, breast cancer 11, cervical cancer 9, leukemia 6, seborrheic keratosis 33, seborrheic dermatitis 28, dermatoheliosis 16, actinic keratosis 14, compound nevi 13, dermatofibroma 9, lentigines 7, non-melanocytic skin cancer 16, basal cell carcinoma 11, squamous cell carcinoma 4, acne 6, verruca vulgaris 5, rosacea 5, melanoma 5, onychodystrophy 5, hyperpigmentation 4, xerosis 4, intertrigo 3, drug reaction hypersensitivity 2, calluses 2, asteatotic dermatitis 2, atopic dermatitis 2, epidermal cyst 2, vitiligo 1, hidradenitis suppurativa 1, prurigo nodularis 1, folliculitis 1, tinea capitis 1, ecchymosis 1, psoriasis 1, petechiae 1, steroid atrophy 1, Schamberg purpura 1, impetigo 1, xerostoma 1, acrochordon 1, telogen effluvium 1 |
| Aslani, 2020(44) | Retrospective, descriptive study | 22 FFA | 42.3 | 100 | Hypothyroidism 9, hypertension 4, allergic rhinitis/ asthma 4, dyslipidemia 3, ischemic heart disease 3, diabetes mellitus/ impaired fasting glucose 3, rheumatoid arthritis 2, lichen planus 2, psychiatric disease 2, iron deficiency anemia 1, duodenal ulcer 1, migraine 1, irritable bowel syndrome 1 |
| Valesky, 2019(80) | Prospective, descriptive study | 12 FFA | 70.3 | 100 | Hypothyroidism 15, lichen planus 55 |
| Tomasini, 2019(124) | Retrospective, descriptive study | 30 EPD | 76 | 26.7 | Hypothyroidism 1, collagenous colitis 1, collagen vascular disease 1, actinic keratosis 6, basal cell carcinoma 2, squamous cell carcinoma 1, discoid lupus erythematosus 1 |
| Secchin, 2019(74) | Retrospective, descriptive study | 16 FFA | 62 | 100 | Systemic lupus erythematosus 5, discoid lupus erythematosus 5, hypertension 8, thyroid disease 6, hypothyroidism 5, Hashimoto thyroiditis 2, hyperparathyroidism 1, lichen planus pigmentosus 5, vitiligo 3, diabetes mellitus 2, psoriasis 1, oral lichen planus 1 |
| Kusano, 2019(58) | Retrospective, descriptive study | 34 FFA | 61.1 | 100 | Nail lichen planus 1, lichen planus pigmentosus 1, hypertension 18, dyslipidemia 14, depression 14, thyroid disease 14, hypothyroidism 11, hepatopathy 4, anxiety 3, diabetes mellitus 2, cardiopathy 2, osteoporosis 1, osteopenia 1, non-melanocytic skin cancer 3, vitiligo 2, systemic lupus erythematosus 1, psoriasis 1 |
| Kanti, 2019(57) | Cross-sectional, descriptive study | 490 FFA | 60 | 95 | Thyroid disease 182, hyperthyroidism 11, hypothyroidism 60, Hashimoto thyroiditis 26, unspecified thyroid disease 85, type 1 diabetes mellitus 4, diabetes mellitus 11, coronary artery disease 30, hypertension 89, dyslipidemia 105, autoimmune disease 35, vitiligo 12, cutaneous lupus erythematosus 7, localized scleroderma 1, alopecia areata 2, rheumatoid arthritis 4, polymyalgia rheumatica 2, systemic sclerosis 2, Sjogren syndrome 1, Sharp syndrome 1, autoimmune hepatitis 1, giant cell arteritis 1, Celiac disease 1, atopy 17, rosacea 9, psoriasis 3, psoriatic arthritis 1, irritable bowel syndrome 3, Crohn disease 1, ulcerative colitis 1 |
| Doche, 2019(111) | Retrospective, descriptive study | 57 AKN | NR | 17.5 | FD 12 |
| Babahosseini, 2019(21) | Retrospective, descriptive study | 261 LPP | 41.5 | 62 | Hypothyroidism 15, lichen planus 55 |
|  |  | 26 FFA | 43 | 88.4 | Hypothyroidism 4, lichen planus 0 |
| Zhang, 2018(83) | Retrospective, descriptive study | 29 FFA | 55.4 | 97 | Thyroid disease 13, hypothyroidism 10, mucocutaneous lichen planus 8, breast cancer 6, psoriasis 2, iritis 1, Sjogren syndrome 1, systemic lupus erythematosus 1, ulcerative colitis 1, vitiligo 1 |
| Strazzulla, 2018(76) | Retrospective, descriptive study | 92 FFA | 55 | 98 | Autoimmune disease 10, hypothyroidism 7, lichen planus 1, psoriasis 2, rosacea 3, endometriosis/ polycystic ovarian syndrome 2, lichen sclerosus et atrophicans 4, anxiety/ depression 5 |
| Pindado-Ortega, 2018(67) | Cross-sectional, descriptive study | 103 FFA | 55.8 | 100 | Rosacea 35, autoimmune disease 10 |
| Miguel-Gómez, 2018(102) | Retrospective, multicenter, descriptive study | 60 FD | 40 | 38.3 | Dyslipidemia 8, hypertension 7, pollen allergy 3, obesity 2, non-Hodgkin lymphoma 1, androgenetic alopecia 14, atopic dermatitis 4, seborrheic dermatitis 2, psoriasis 2, hyperhidrosis 1, alopecia areata 1, interstitial granulomatous dermatitis 1, hidradenitis suppurativa 1, basal cell carcinoma 1, vitiligo 1, acne 1 |
| Kurt, 2018(27) | Retrospective, descriptive study | 40 LPP | 43.7 | 62.5 | Diabetes mellitus 3, hypertension 4, coronary artery disease 5, dyslipidemia 3, thyroid disease 8 |
|  |  | 15 FFA | 62.2 | 100 | Diabetes mellitus 3, hypertension 6, coronary artery disease 7, dyslipidemia 3, thyroid disease 1 |
| Imhof, 2018(54) | Retrospective, descriptive study | 148 FFA | 62.1 | 100 | Thyroid disease 68, dyslipidemia 67, hypothyroid 66, atopy 65, hypertension 55, depression 41, seborrheic dermatitis 27, obesity 27, osteopenia 25, osteoporosis 23, rosacea 23, impaired fasting glucose 20, hypertriglyceridemia 18, vitamin D deficiency 16, anemia 15, Hashimoto thyroiditis 12, endometriosis 11, psoriasis 11, obstructive sleep apnea 9, inflammatory bowel disease 8, acne 8, lichen sclerosus 8, Sjogren syndrome 6, systemic lupus erythematosus 5, diabetes mellitus 5, vitiligo 4, hirsutism 3, celiac 3, Graves disease 2, scleroderma 2, polycystic ovarian syndrome 2, rheumatoid arthritis 2, type 1 diabetes mellitus 1, lichen planus 27, oral lichen planus 16, vulvar lichen planus 15 |
| Heppt, 2018(53) | Retrospective, descriptive study | 72 FFA | 64 | 97.2 | Hypothyroidism 21, diabetes mellitus 2, hyperparathyroidism 1, polyarthritis 6, giant cell arteritis 1, hypertension 21, psoriasis 2, mucosal lichen planus 3, alopecia areata 1, melanoma 4, non-melanocytic skin cancer 2, androgenetic alopecia 25, leukemia 1, breast cancer 3, lung cancer 1 |
| Gkini, 2018(51) | Retrospective, descriptive study | 40 FFA | 65.9 | 100 | Androgenetic alopecia 15, LPP 3, lichen planus 4, Hashimoto thyroiditis 1, psoriasis 1, eczema 1, fatty liver 1, angular cheilitis 1, perioral dermatitis 1, seborrheic dermatitis 1, breast cancer 1, hypertrichosis 1 |
| Starace, 2017(123) | Retrospective, descriptive study | 20 EPD | 59.4 | 65 | Androgenetic alopecia 12, thyroiditis 3, rheumatoid arthritis 1 |
| Na, 2017(114) | Retrospective, descriptive study | 17 AKN | 41.1 | 0 | Metabolic syndrome 3, diabetes mellitus 2, renal cell carcinoma 1, hypertension 1, dyslipidemia 1, gynecomastia 1 |
| East-Innis, 2017(112) | Retrospective, case-control study | 43 AKN | 25.4 | 12 | Seborrheic dermatitis 11, acne 8, pseudofolliculitis barbae 6, chronic scalp folliculitis 5, FD 3 |
| Suchonwanit, 2016(98) | Cross-sectional, descriptive study | 38 CCCA | 45.8 | 100 | Acne 25, keloid 12, bacterial infection 2, tinea capitis 1, ringworm 4, vaginal yeast infection 23, contact dermatitis 6, seborrheic dermatitis 22, diabetes mellitus 4, thyroid disease 3 |
| Badaoui, 2016(106) | Retrospective, descriptive study | 51 DC | 26.6 | 2 | Hidradenitis suppurativa 6, acne conglobata 8 |
| Vañõ-Galván, 2015(104) | Retrospective, multicenter, descriptive study | 82 FD | 35 | 36.6 | Hypertension 7, dyslipidemia 4, thyroiditis 2, atopic dermatitis 15, hidradenitis suppurativa 2, androgenetic alopecia 28 |
| Özcan, 2015(31) | Retrospective, descriptive study | 25 LPP | 49.8 | 72 | Hashimoto thyroiditis 3, type 1 diabetes mellitus 1, lichen planus pigmentosus 1, mucosal lichen planus 2 |
| Meinhard, 2014(30) | Retrospective, descriptive study | 71 LPP | NP | 83.1 | Coronary heart disease 2, hypertension 25, dyslipidemia 9, diabetes mellitus 3, lichen planus 4, alopecia areata 1, vitiligo 0, thyroid disease 7, depression 5, iron deficiency 3, viral hepatitis 1, seborrheic dermatitis 3, chronic obstructive pulmonary disease 2, breast cancer 2 |
|  |  | 32 FFA | NP | 96.9 | Coronary heart disease 3, hypertension 12, dyslipidemia 3, diabetes mellitus 1, lichen planus 0, alopecia areata 1, vitiligo 2, thyroid disease 10, depression 0, iron deficiency 1, viral hepatitis 0, seborrheic dermatitis 0, chronic obstructive pulmonary disease 0, breast cancer 0 |
| Bunagan, 2014(100) | Retrospective, descriptive study | 23 FD | NP | 30.4 | Psoriasis 2, dyslipidemia 2, coronary heart disease 1, hypothyroidism 1, leukemia 1, diabetes mellitus 1, rheumatoid arthritis 1, hypertension 1, FFA 1 |
| Banka, 2014(45) | Retrospective, descriptive study | 62 FFA | NP | 98 | LPP 10, mucocutaneous lichen planus 1, genital lichen planus 1, androgenetic alopecia 43, alopecia areata 8, psoriasis 2, vitiligo 1, systemic lupus erythematosus 2, discoid lupus erythematosus 2, rheumatoid arthritis 4, polymyositis 1, hypothyroidism 13, Hashimoto thyroiditis 3 |
| Shah, 2010(97) | Retrospective, descriptive study | 185 CCCA | 53.7 | 100 | Diabetes mellitus 44, dyslipidemia 65, hyperthyroidism 1, hypothyroidism 6, other thyroid disease 4, hypertension 95 |
| Tan, 2009(78) | Retrospective, descriptive study | 18 FFA | 55.5 | 100 | Mucocutaneous lichen planus 3, thyroid disease 2, vitiligo 1 |

AKN, acne keloidalis nuchae; CCCA, central centrifugal cicatricial alopecia; DC, dissecting cellulitis; EPD, erosive pustular dermatosis; FD, folliculitis decalvan; FAPD, fibrosing alopecia in a pattern distribution; FFA, frontal fibrosing alopecia; LPP, lichen planopilaris
