# Supplementary material for: Comorbidities in primary cicatricial alopecia: a systematic review and meta-analysis
Source: Front Immunol. 2025 Aug 29;16:1516407. doi: 10.3389/fimmu.2025.1516407 (PMC12426186; doi:10.3389/fimmu.2025.1516407)
Supplement: Supplementary file 14 [file Table6.docx]

**Supplementary** **Table 6.** Characteristics and main findings of cohort studies that included patient with primary cicatricial alopecia

| **Study** | **Study source and design** | **Study population, N** | **Mean age, y/ female, %** | **Control, N** | **Mean age, y/ female, %** | **Main findings** |
| --- | --- | --- | --- | --- | --- | --- |
| Kim, 2024(125) | Nationwide, longitudinal cohort study, National Health Insurance Service database | 573 LPP/FFA | 36.4/ 36.4* | 319,440 age- and gender-matched normal controls | 36.4/ 36.4* | - CVD aHR^†^ 1.18 (0.69–2.04) - CHD aHR^†^ 1.93 (1.07–3.49) - Stroke aHR^†^ 0.60 (0.23–1.60) |
|  |  | 129 PPB | 36.4/ 36.4* | 319,440 age- and gender-matched normal controls | 36.4/ 36.4* | - CVD aHR^†^ 2.40 (0.76–7.42) - CHD aHR^†^ 2.91 (0.74–11.8) - Stroke aHR^†^ 1.43 (0.20–10.2) |
|  |  | 3368 FD | 36.4/ 36.4* | 319,440 age- and gender-matched normal controls | 36.4/ 36.4* | - CVD aHR^†^ 1.29 (1.04–1.61) - CHD aHR^†^ 1.25 (0.93–1.69) - Stroke aHR^†^ 1.39 (1.05–1.84) |
|  |  | 3137 DC | 36.4/ 36.4* | 319,440 age- and gender-matched normal controls | 36.4/ 36.4* | - CVD aHR^†^ 1.05 (0.80–1.38) - CHD aHR^†^ 1.10 (0.77–1.57) - Stroke aHR^†^ 1.02 (0.72–1.47) |
| Shavit, 2023(119) | Retrospective, population-based cohort study, Clalit Health Services database | 2677 AKN | 34.5/ 4.3 | 13,190 age-, gender-, and ethnicity-matched normal controls | 34.5/ 4.3 | - Gout aHR^‡^ 2.34 (1.29–4.22) - Gout aHR^‖^ 1.39 (0.73–2.65) |
| Valdman-Grinshpoun, 2021(120) | Retrospective, population-based cohort study, Clalit Health Services database | 2677 AKN | 34.5/ 4.3 | 13,190 age-, gender-, and ethnicity-matched normal controls | 34.5/ 4.3 | - Hypothyroidism HR 1.85 (1.24–2.78) - Hypothyroidism aHR^§^ 1.72 (1.03–2.89) - Hyperthyroidism HR 1.55 (0.57–4.22) - Hyperthyroidism aHR^§^ 1.92 (0.59–6.21) |

aHR, adjusted hazard ratio; CHD, coronary heart disease; CVD, cardiovascular disease; HR, hazard ratio
* Data from the whole cohort

^†^ Adjusting for age, sex, household income, smoking, alcohol intake, physical activity, systolic blood pressure, fasting serum glucose, total cholesterol, and Charlson comorbidity index

^‡^ Adjusting for age, sex, and ethnicity

^‖^ Adjusting for body mass index, diabetes mellitus, hypertension, and dyslipidemia

^§^ Adjusting for age, gender, ethnicity (Jews vs. Arabs), and origin (African vs. non-Africans)
