# Supplementary material for: Comorbidities in primary cicatricial alopecia: a systematic review and meta-analysis
Source: Front Immunol. 2025 Aug 29;16:1516407. doi: 10.3389/fimmu.2025.1516407 (PMC12426186; doi:10.3389/fimmu.2025.1516407)
Supplement: Supplementary file 15 [file Table7.docx]

**Supplementary** **Table 7.** The quality assessment of studies included in the systematic review

| **Study** | **Case** | | **Control** | | **Comparability** | | **Exposure** | | | **Overall quality score** |
| --- | --- | --- | --- | --- | --- | --- | --- | --- | --- | --- |
|  | **Definition (*)** | **Represen-tativeness (*)** | **Selection (*)** | **Definition (*)** | **Age/ gender (*)** | **Additional (*)** | **Ascertainment (*)** | **Same method (*)** | **Non-response rate (*)** |  |
| Yu, 2024(20) | - | * | * | * | * | - | * | * | * | 7 |
| Joshi, 2024(86) | - | * | * | * | * | * | * | * | * | 8 |
| Sung, 2023(17) | - | * | * | * | * | * | * | * | * | 8 |
| Leung, 2023(88) | * | * | * | - | * | * | * | * | * | 8 |
| Joshi, 2023(13) | - | * | * | - | * | * | * | * | * | 7 |
| Jafari, 2023(85) | * | * | - | * | * | * | * | * | * | 8 |
| Tran, 2022(105) | - | * | - | * | - | - | * | * | * | 5 |
| Roche, 2022(91) | * | * | - | * | * | * | * | * | * | 8 |
| Porriño-Bustamante, 2022(40) | * | * | - | * | * | - | * | * | * | 7 |
| Nasimi, 2022(15) | * | * | - | * | * | * | * | * | * | 8 |
| Bazotti, 2022(34) | * | - | - | * | * | * | * | * | * | 7 |
| Arasu, 2022(33) | - | - | - | * | * | - | * | * | * | 5 |
| Trager, 2021(19) | * | * | - | * | - | - | * | * | * | 6 |
| Samrao, 2021(92) | * | * | - | * | * | * | * | * | * | 8 |
| Ramos, 2021(41) | * | * | - | * | * | - | * | * | * | 7 |
| McKenzie, 2021(89) | - | * | - | - | - | - | * | * | * | 4 |
| Leecharoen, 2021(37) | * | - | - | * | * | - | * | * | * | 6 |
| Kridin, 2021(109) | * | * | * | - | * | * | * | * | * | 8 |
| Conic, 2021(10) | - | * | * | * | - | - | * | * | * | 6 |
| Brown-Korsah, 2021(99) | * | * | - | * | * | * | * | * | * | 8 |
| Saka, 2020(118) | * | * | - | * | * | - | * | * | * | 7 |
| Rudnicka, 2020(42) | * | - | - | * | * | - | * | * | * | 6 |
| Narasimman, 2020(90) | * | * | - | * | * | * | * | * | * | 8 |
| Manatis-Lornell, 2020(14) | * | * | - | * | * | * | * | * | * | 8 |
| Donati, 2020(36) | - | * | * | * | - | - | * | * | * | 6 |
| Porriño-Bustamante, 2019(39) | * | - | - | * | * | - | * | * | * | 6 |
| Moreno-Arrones, 2019(38) | * | * | - | * | * | - | * | * | * | 7 |
| Fertig, 2018(12) | * | * | - | * | * | * | * | * | * | 8 |
| Dina, 2018(84) | - | * | * | * | * | * | * | * | * | 8 |
| Conic, 2018(11) | * | * | - | * | * | * | * | * | * | 8 |
| Buendía-Castaño, 2018(35) | * | * | - | * | * | - | * | * | * | 7 |
| Brankov, 2018(9) | * | * | - | * | * | * | * | * | * | 8 |
| Nguyen, 2016(16) | * | * | - | * | * | - | * | * | * | 7 |
| Aldoori, 2016(32) | * | * | * | * | * | - | * | * | * | 8 |
| Toossi, 2015(18) | * | * | - | * | * | - | * | * | * | 7 |
| Atanaskova Mesinkovska, 2014(8) | * | * | - | * | * | - | * | * | * | 7 |
| Kyei, 2011(87) | * | * | * | * | * | - | * | * | * | 8 |

| **Study** | **Selection** | | | | **Comparability** | | **Exposure** | | | **Overall quality score** |
| --- | --- | --- | --- | --- | --- | --- | --- | --- | --- | --- |
|  | **Represen-tativeness (*)** | **Non-exposed selection (*)** | **Ascertainment of exposure (*)** | **Outcome not at the start (*)** | **Age/gender (*)** | **Additional (*)** | **Ascertainment (*)** | **Follow-up duration (*)** | **Adequacy of follow-up (*)** |  |
| Kim, 2024(125) | * | * | * | * | * | * | * | * | * | 9 |
| Shavit, 2023(119) | * | * | * | * | * | * | * | * | * | 9 |
| Valdman-Grinshpoun, 2021(120) | * | * | * | * | * | * | * | * | * | 9 |

| **Study** | **Sample** | | | **Exposure** | **Comparability** | | **Outcome** | | **Overall quality score** |
| --- | --- | --- | --- | --- | --- | --- | --- | --- | --- |
|  | **Represen-tativeness (*)** | **Sample size (*)** | **Non-respondents (*)** | **Ascertainment (**)** | **Age (*)** | **Additional (*)** | **Assessment (*)** | **Statistics (*)** |  |
| Sarkis, 2024(103) | * | * | * | ** | * | * | * | * | 9 |
| Lyakhovitsky, 2024(29) | * | * | * | ** | * | * | * | * | 9 |
| Lobato-Berezo, 2024(113) | * | - | * | * | * | * | * | * | 7 |
| Carrascoza, 2024(23) | * | * | * | ** | * | * | * | * | 9 |
| Xavier de Brito, 2023(82) | * | * | * | * | * | * | * | * | 8 |
| Verma, 2023(81) | * | - | * | ** | * | * | * | * | 8 |
| Shamloul, 2023(122) | * | - | * | ** | - | - | * | * | 6 |
| Saceda-Corralo, 2023(71) | * | * | * | ** | * | * | * | * | 9 |
| Parker, 2023(115) | * | - | * | ** | * | * | * | * | 8 |
| Oulad Ali, 2023(65) | * | - | * | * | * | * | * | * | 7 |
| Onamusi, 2023(96) | * | * | * | ** | * | * | * | * | 9 |
| Lyakhovitsky, 2023(101) | * | * | * | ** | * | * | * | * | 9 |
| Jackson, 2023(95) | * | * | * | ** | * | * | * | * | 9 |
| Gharaei Nejad, 2023(26) | * | - | * | ** | * | * | * | * | 8 |
| García, 2023(50) | * | * | * | ** | * | * | * | * | 9 |
| Carmona-Rodríguez, 2023(46) | * | * | * | ** | * | * | * | * | 9 |
| Balazic, 2023(94) | * | * | * | ** | * | * | * | * | 9 |
| Umar, 2022(117) | * | * | * | * | * | - | * | * | 7 |
| Starace, 2022(75) | * | * | * | ** | * | * | * | * | 9 |
| Salas-Callo, 2022(73) | * | - | * | * | * | * | * | * | 7 |
| Rocha, 2022(69) | * | - | * | ** | * | * | * | * | 8 |
| Moussa, 2022(64) | * | - | * | ** | * | * | * | * | 8 |
| Melo, 2022(108) | * | - | * | ** | * | * | * | * | 8 |
| Lobato-Berezo, 2022(59) | * | - | * | ** | * | * | * | * | 8 |
| Jiang, 2022(56) | * | * | * | ** | * | * | * | * | 9 |
| Dorgham, 2022(49) | * | - | * | ** | * | * | * | * | 8 |
| Doche, 2022(25) | * | - | * | * | * | * | * | * | 7 |
| Doche, 2022(48) | * | - | * | ** | * | * | * | * | 8 |
| Collins, 2022(47) | * | - | * | ** | - | - | * | * | 6 |
| Ali, 2022(93) | * | - | * | ** | * | * | * | * | 8 |
| Uzunçakmak, 2021(79) | * | - | * | ** | * | * | * | * | 8 |
| Umar, 2021(116) | * | * | * | ** | * | * | * | * | 9 |
| Rossi, 2021(70) | * | - | * | ** | * | * | * | * | 8 |
| Pindado-Ortega, 2021(68) | * | * | * | ** | * | * | * | * | 9 |
| Michelerio, 2021(121) | * | - | * | ** | * | * | * | * | 8 |
| Melo, 2021(62) | * | - | * | ** | * | * | * | * | 8 |
| Jerjen, 2021(55) | * | - | * | ** | * | * | * | * | 8 |
| Grassi, 2021(52) | * | * | * | ** | * | * | * | * | 9 |
| Feng, 2021(107) | * | - | * | * | * | * | * | * | 7 |
| Conic, 2021(24) | * | - | * | * | * | * | * | * | 7 |
| Cantwell, 2021(22) | * | - | * | ** | * | * | * | * | 8 |
| Adotama, 2021(43) | * | * | * | ** | * | * | * | * | 9 |
| Suchonwanit, 2020(77) | * | - | * | ** | * | * | * | * | 8 |
| Saceda-Corralo, 2020(72) | * | - | * | ** | * | * | * | * | 8 |
| Panchaprateep, 2020(66) | * | - | ** | ** | * | * | * | * | 8 |
| McSweeney, 2020(61) | * | * | * | ** | * | * | * | * | 9 |
| Maldonado Cid, 2020(60) | * | - | * | ** | * | * | * | * | 8 |
| Larkin, 2020(28) | * | * | * | ** | * | * | * | * | 9 |
| Aslani, 2020(44) | * | - | * | ** | * | * | * | * | 8 |
| Valesky, 2019(80) | * | - | * | ** | * | * | * | * | 8 |
| Tomasini, 2019(124) | * | - | * | ** | * | * | * | * | 8 |
| Secchin, 2019(74) | * | - | * | ** | * | * | * | * | 8 |
| Kusano, 2019(58) | * | - | * | ** | * | * | * | * | 8 |
| Kanti, 2019(57) | * | * | * | ** | * | * | * | * | 9 |
| Doche, 2019(111) | * | - | * | ** | - | - | * | * | 6 |
| Babahosseini, 2019(21) | * | * | * | ** | * | * | * | * | 9 |
| Zhang, 2018(83) | * | - | * | ** | * | * | * | * | 8 |
| Strazzulla, 2018(76) | * | - | * | ** | * | * | * | * | 8 |
| Pindado-Ortega, 2018(67) | * | * | * | ** | * | * | * | * | 9 |
| Miguel-Gómez, 2018(102) | * | - | * | ** | * | * | * | * | 8 |
| Kurt, 2018(27) | * | - | * | ** | * | * | * | * | 8 |
| Imhof, 2018(54) | * | * | * | ** | * | * | * | * | 9 |
| Heppt, 2018(53) | * | - | * | ** | * | * | * | * | 8 |
| Gkini, 2018(51) | * | - | * | ** | * | * | * | * | 8 |
| Starace, 2017(123) | * | - | * | ** | * | * | * | * | 8 |
| Na, 2017(114) | * | - | * | ** | * | * | * | * | 8 |
| East-Innis, 2017(112) | * | - | * | ** | * | * | * | * | 8 |
| Suchonwanit, 2016(98) | * | - | * | ** | * | * | * | * | 8 |
| Badaoui, 2016(106) | * | - | * | ** | * | * | * | * | 8 |
| Vañõ-Galván, 2015(104) | * | - | * | ** | * | * | * | * | 8 |
| Özcan, 2015(31) | * | - | * | ** | * | * | * | * | 8 |
| Meinhard, 2014(30) | * | - | * | ** | - | - | * | * | 6 |
| Bunagan, 2014(100) | * | - | * | ** | - | - | * | * | 6 |
| Banka, 2014(45) | * | - | * | ** | - | - | * | * | 6 |
| Shah, 2010(97) | * | * | * | ** | * | * | * | * | 9 |
| Tan, 2009(78) | * | - | * | ** | * | * | * | * | 8 |
